# Dedoping of Intraband Silver Selenide Colloidal Quantum Dots through Strong Electronic Coupling at Organic/Inorganic Hybrid Interfaces

**DOI:** 10.1021/acs.cgd.3c01474

**Published:** 2024-03-22

**Authors:** Håvard Mo̷lnås, Shlok Joseph Paul, Michael R. Scimeca, Navkawal Mattu, Jiaqi Zuo, Nitika Parashar, Letian Li, Elisa Riedo, Ayaskanta Sahu

**Affiliations:** Department of Chemical and Biomolecular Engineering, Tandon School of Engineering, New York University, Brooklyn, New York 11201, United States

## Abstract

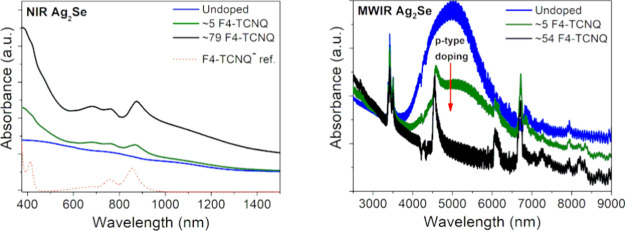

Colloidal quantum dot (CQD) infrared (IR) photodetectors can be fabricated and operated with larger spectral tunability, fewer limitations in terms of cooling requirements and substrate lattice matching, and at a potentially lower cost than detectors based on traditional bulk materials. Silver selenide (Ag_2_Se) has emerged as a promising sustainable alternative to current state-of-the-art toxic semiconductors based on lead, cadmium, and mercury operating in the IR. However, an impeding gap in available absorption bandwidth for Ag_2_Se CQDs exists in the short-wave infrared (SWIR) region due to degenerate doping by the environment, switching the CQDs from intrinsic interband semiconductors in the near-infrared (NIR) to intraband absorbing CQDs in the mid-wave infrared (MWIR). Herein, we show that the small molecular p-type dopant 2,3,5,6-tetrafluoro-7,7,8,8-tetracyanoquinodimethane (F4-TCNQ) can be used to extract electrons from the 1S_e_ state of MWIR active Ag_2_Se CQDs to activate their intrinsic energy gap in the SWIR window. We demonstrate quenching of the MWIR Ag_2_Se absorbance peak, shifting of nitrile vibrational peaks characteristic of charge-neutral F4-TCNQ, as well as enhanced CQD absorption around ∼2500 nm after doping both in ambient and under air-free conditions. We elucidate the doping mechanism to be one that involves an integer charge transfer akin to doping in semiconducting polymers. These indications of charge transfer are promising milestones on the path to achieving sustainable SWIR Ag_2_Se CQD photodetectors.

## Abstract 2

Localized surface doping using a strong electron acceptor was explored to expand the photoactive range of environmentally benign silver selenide colloidal quantum dots into the short-wave infrared range currently inaccessible by hot injection synthesis. Quenching of the mid-wave infrared absorbance peak and enhanced absorption around 2.5 μm indicate charge transfer and increased photoactivity in previously inactive ranges for this semiconductor.

## 1 Introduction

As photodetectors operating in the vast IR spectrum are being utilized in a growing number of markets,^1^ absorber tunability, sustainability, and array fabrication costs are under increased scrutiny.^2,3^ State-of-the-art bulk semiconductors like indium gallium arsenide (In_*x*_Ga_1–*x*_As) and mercury cadmium telluride (Hg_1–*x*_Cd_*x*_Te/MCT) have been reliable workhorses for decades, but require complex and expensive fabrication techniques including careful lattice matching to enable bandgap tunability.^4−6^ Alternatively, semiconductor CQDs can be easily synthesized at relatively low temperatures and provide high spectral tunability through size control and surface chemistry.^7−9^ However, state-of-the-art IR active materials, both in bulk and as CQDs, are typically composed of toxic elements such as lead (Pb), mercury (Hg), arsenic (As), and cadmium (Cd), raising concerns for usage, especially in terrestrial and nonmilitary applications.^10−12^

In this context, Ag_2_Se is a promising alternative IR semiconductor, having a narrow bulk bandgap of ∼0.05–0.18 eV.^13^ As nanoparticles, Ag_2_Se demonstrates extensive photoresponse tunability from the NIR to the long-wave infrared (LWIR) region of the electromagnetic spectrum by varying particle diameter in the range of 2.6–27 nm.^13−21^ The NIR response emerges from the interband energy transition (“the band gap”, between the 1S_h_ and 1S_e_ states), while the MWIR and LWIR response is perceived to be the result of an intraband energy transition from the 1S_e_ to 1P_e_ state.^21−27^ Intraband transitions in IR CQDs have been explored more extensively in the Hg chalcogenide literature,^28−33^ suggesting that energy level filling and stable n-type doping is a result of band edge positions relative to the environmental Fermi level, *E*_F,env_.^34−37^

Originally, separate synthesis approaches were required for small (<5 nm) and large (>5 nm) Ag_2_Se CQDs, following thiol-based and amine-based routes, respectively.^13−15,23^ Recently, we have reported a thiol-based synthesis route capable of fabricating both NIR and MWIR Ag_2_Se CQDs.^23^ Following this protocol using controlled growth conditions, at a certain particle size typically around 5 nm, particles transition from displaying interband absorbance in the NIR to showing intraband absorbance in the MWIR. However, we were not able to achieve CQDs that demonstrate significant optical absorbance in the SWIR window through this route, and in general, limited reports exist for SWIR Ag_2_Se CQD photodetectors.^2,27^ The crossover from interband to intraband absorption renders the wavelength range 2–4 μm effectively inaccessible by hot-injection synthesis,^20,22,24^ however, an intraband band gap as high as 0.39 eV/3.1 μm has been reported following a cation exchange route.^25^ This phenomenon of doping by the environment, while interesting, unfortunately, creates a spectral gap for Ag_2_Se CQDs in the SWIR and thus limits its application as a truly wide-range nontoxic IR absorber.

Based on a two-band k.p model with Kane parameter 8.7,^23,38^ the interband energy gap of Ag_2_Se CQDs having a certain intraband gap can be predicted. For 5.2 nm diameter, MWIR active Ag_2_Se CQDs with an intraband energy gap of ∼5 μm/0.248 eV, the interband gap would be in the SWIR (∼2 μm/0.62 eV), as demonstrated in Figure S.1 in Section 1 of the Supporting Information (SI). We hypothesize that efficient extraction of electrons from the 1S_e_ state would enable the interband energy gap for the absorption of SWIR photons. Electron extraction could be obtained through extrinsic foreign atom doping; however, such doping in CQDs is not trivial and could easily lead to alloying and/or phase segregation while significantly modifying the inherent CQD band structure and impacting the desired optoelectronic properties.^39^ Especially for Ag_2_Se CQDs that demonstrate IR photoresponse owing to the metastable tetragonal crystal structure, we postulate that spatial separation of host and dopant atoms would be ideal to achieve the high doping (dedoping) levels required to remove the excess electrons by environmental doping.

For the proposed study, we draw inspiration from a commonly employed small molecular p-type dopant in organic semiconductors, F4-TCNQ.^40−42^ Serving as an excellent electron acceptor with a deep LUMO level at −5.2 eV relative to vacuum, F4-TCNQ can introduce p-type doping in semiconductors with a preferable band energy alignment. This has been demonstrated for organic polymers,^43−47^ organic–inorganic halide perovskites,^48,49^ epitaxial graphene,^50−52^ as well as Te NWs and other inorganic QD semiconductors.^54−58^

Zhao et al.^54^ doped PbS CQDs of various sizes with F4-TCNQ and demonstrated fractional charge transfer from the dopant to the CQD through an orbital mixing mechanism. A reversible absorption blue-shift of PbS CQDs was observed, indicating a CQD/F4-TCNQ electronic interaction. Additionally, improved performance of CdSe/CdSe nanorod-based LEDs has been observed through F4-TCNQ doping,^57^ and electron transfer from CQDs to a molecular dopant has also been shown for the related, but less polar molecule tetracyanoquinodimethane (TCNQ).^55,56,58^

A challenge with molecular doping, particularly in organic semiconductors such as poly(3-hexylthiophene) (P3HT), is the low doping efficiency.^59^ In some cases, a dopant amount of up to 35–50% F4-TCNQ is necessary to achieve sufficient change in electrical conductivity in organic material.^60^ Using traditional substitutional doping with such a high concentration of foreign atoms for inorganic semiconductors can distort the crystal structure of the host materials, particularly for CQDs. However, we hypothesize that by confining the dopant to the CQD surface, efficient doping can be achieved through much lower dopant concentrations. In a recent study, we demonstrate maximum conductivity in Te nanowires through 20% surface coverage with F4-TCNQ, equating to a total doping concentration of ∼0.5%.

Building on these promising results, in this work, we therefore introduce F4-TCNQ as a molecular dopant for Ag_2_Se CQDs and investigate the changes in optical absorption in the visible, NIR, SWIR, and MWIR resulting from different doping parameters under ambient and air-free conditions. We demonstrate successful reduced n-type doping of Ag_2_Se CQDs through the presence of optical signatures of the F4-TCNQ^–^ monoanion after doping of tetragonal NIR and MWIR Ag_2_Se CQDs, quenching of the Ag_2_Se intraband MWIR absorption by F4-TCNQ, and the appearance of an absorption feature at ∼1900 nm, matching the interband transition expected from a two-band k.p model. Investigating the doping mechanism, we conclude, based on the spectroscopic studies, that the doping occurs via a ground-state integer charge transfer rather than a charge-transfer complex formation route. These results represent important first steps in order to access the SWIR range utilizing Ag_2_Se CQDs, bridging the current energy gap for this promising nontoxic IR active semiconductor.

## 2 Materials and Methods

### 2.1 Chemicals

Silver nitrate (AgNO_3_, ≥99.9%), selenium pellets (<5 mm, ≥99.99%), trioctylphosphine (TOP, 97%), trioctylphosphine oxide (TOPO, technical grade, ≥90%), oleylamine (OlAm, technical grade, 70%), 1-dodecanethiol (DDT, ≥98%), toluene (C_6_H_5_CH_3_, anhydrous, 99.8%), hexane (C_6_H_14_, anhydrous, 95%), isopropyl alcohol (IPA, anhydrous, 99.5%), octane (C_8_H_18_, anhydrous, ≥99%), 1,2-ethanedithiol (EDT, technical grade, ≥90%), chloroform (CHCl_3_, anhydrous, ≥99%), and hydrochloric acid (HCl, puriss. 24.5–26.0%) were purchased from Sigma-Aldrich. 2,3,5,6-Tetrafluoro-7,7,8,8-tetracyanoquinodimethane (F4-TCNQ, ≥98.0%) was purchased from TCI. Acetone (C_3_H_6_O, extra dry, 99.8%) and 4-*tert*-butyl-toluene (TBT, 96%) were purchased from Acros Organics. Ethanol (reagent grade), toluene (reagent grade), hexane (reagent grade), isopropanol (reagent grade), and acetone (reagent grade) were purchased from Greenfield Global. Deionized water (DI water, 18 MΩ) was fabricated in-house in a Millipore Milli-Q Integral 3 Water Purification System. All chemicals were used as received without further purification.

### 2.2 NIR Ag_2_Se CQD Synthesis

NIR-active (∼3 nm) Ag_2_Se CQDs were synthesized based on an adaptation of routes described in our previous reports.^19,23^ Briefly, 8.75 mL of DDT was mixed with 250 mL of TBT, and 2.565 g of AgNO_3_ was dissolved in 250 mL of DI water in two separate containers. After 30 min stirring, the solutions were mixed together and stirred in the dark for 2 h, forming a milky yellow solution. Upon 20 min settling in a separatory funnel, phase separation was observed with the milky Ag*DDT complex phase on top. The bottom aqueous phase was removed and discarded. The Ag*DDT precursor was transferred to a four-neck flask, connected to a Schlenk line, and purged with nitrogen (N_2_) for 30 min. The solution was then heated to 170 °C, at which point 32.5 mL of 1.0 M TOP:Se diluted with 10 mL of TOP was injected rapidly into the flask. After 60 min growth at 170 °C, the reaction was quenched using a water bath, and 120 mL of ethanol was added to precipitate the CQDs through 5 min centrifugation at 6000 rpm. The precipitate was redispersed in toluene and centrifuged to enable the removal of precipitated impurities. Ethanol was added to the supernatant, and the solution was centrifuged for 5 min at 6000 rpm to precipitate the final product. The CQDs were dried under vacuum and stored in the dark in a N_2_-filled glovebox until needed.

### 2.3 MWIR Ag_2_Se CQD Synthesis

MWIR-active (∼5.2 nm) Ag_2_Se CQDs were synthesized in accordance with Sahu et al.^13^ with slight adaptations.

1.0 M TOP-Ag solution and 1.0 M TOP-Se solution were prepared by dissolving 1.699 g of AgNO_3_ and 790 mg of crushed Se pellets in 10 mL of 97% TOP, respectively, stirring overnight in a N_2_-filled glovebox.

15.6 g of TOPO and 13.2 mL of OlAm were degassed at 65 °C in a 250 mL three-neck flask connected to a Schlenk line. When degassing was completed, 8 mL 1.0 M TOP-Se was injected under the N_2_ atmosphere, before the temperature was raised to 170 °C. At 170 °C, 8 mL 1.0 M TOP-Ag was injected rapidly to initiate nucleation, and the CQDs were grown at 160 °C for 12 min. At the end of the growth period, the heating mantle was removed, and the solution was quenched using a water bath and through the addition of 40 mL of anhydrous toluene.

The synthesized Ag_2_Se CQDs were precipitated with ethanol, and the pellet was resuspended in toluene. The toluene solution was centrifuged to remove any metallic silver formed during the synthesis. The supernatant containing the Ag_2_Se CQDs was filtered through a 0.2 μm polytetrafluoroethylene (PTFE) syringe filter before precipitation with ethanol and drying under a vacuum for 10 min. The CQDs were stored under N_2_ atmosphere until further use.

### 2.4 CQD Doping by F4-TCNQ

As-synthesized and ligand-exchanged Ag_2_Se CQD thin films were treated with F4-TCNQ in a dripping or soaking process in ambient or under air-free conditions. For air-free samples, the F4-TCNQ treatment took place in a N_2_-filled glovebox, and the sample was encapsulated in an air-free holder. In the dripping process, F4-TCNQ dissolved at a certain concentration (typically 1 mg/mL = 3.6 mM, but 0.36, 0.58, and 0.72 mM were also utilized) in IPA, acetone or chloroform was added dropwise to the samples, and the absorbance spectra were recorded between each drop after complete drying. Achieved F4-TCNQ concentrations were determined as discussed in Section 2 of the SI and visualized in Figures S.2 and S.3.

In the soaking process, the samples were soaked for a certain amount of time (typically 10–60 s) in F4-TCNQ dissolved at a certain concentration in IPA, acetone, or chloroform and allowed to dry, before recording absorbance spectra. A resulting blue tint was observed in the drop-doped area of the sample thin films after drying but not after soaking in the same solution. The fresh F4-TCNQ solution in IPA displayed a yellow color and turned blue over the course of a few days in ambient conditions and under air-free conditions.

### 2.5 UV–Vis–NIR Spectroscopy

A CARY 5000 UV–vis-NIR spectrometer was utilized to measure thin-film absorbance in the range 200–3000 nm. Ag_2_Se thin films were prepared through drop-casting of 30 mg/mL Ag_2_Se:TOP in 9:1 hexane:octane solution onto Ø12.7 and Ø32 mm calcium fluoride (CaF_2_) windows from Pike Technologies Inc. Clean CaF_2_ windows were used for background correction. For ligand exchanged samples, the entire substrate with the thin film was soaked for 15 s in a 0.02 vol % EDT/HCl in IPA solution and rinsed for 5 s in a neat IPA solution. For air-free measurements, sample preparation took place in an N_2_-filled glovebox, and the sample was enclosed in an air-free holder during the absorbance measurements. HCl was not added to the ligand exchange solution inside the glovebox.

### 2.6 Fourier Transform Infrared Spectroscopy (FTIR)

FTIR spectra in the range 4000–1100 cm^–1^ were recorded using a Nicolet 6700 FTIR spectrometer. Ag_2_Se CQD thin-film samples were prepared in the same way as for UV–vis spectroscopy, as discussed above. Clean CaF_2_ windows were used for background correction.

### 2.7 X-ray Diffraction (XRD)

A Bruker AXS D8 Discover GADDS XRD microdiffractometer equipped with a Cu–Kα source (λ = 1.5405 Å) was utilized to collect wide-angle X-ray diffractograms of Ag_2_Se CQD thin films on 1 × 1 cm Si substrates. The thin films were fabricated in ambient conditions through drop-casting of 30 mg/mL Ag_2_Se:TOP CQD in 9:1 hexane:octane ink onto the substrates. For ligand exchanged samples, the entire Si substrate with the thin film was soaked for 15 s in 0.02 vol % EDT/HCl in IPA followed by 5 s rinse in neat IPA.

### 2.8 Kelvin Probe Force Microscopy (KPFM)

NIR Ag_2_Se:EDT CQD samples were deposited onto clean 10 × 10 mm gold substrates using the same deposition and preparation protocols as described above. Fermi level measurements were performed with the Frequency-Modulated Kelvin Probe Force Microscopy (FM-KPFM) mode on the Bruker Multimode 8 Atomic Force Microscope (AFM) and by using silicon tip on silicon nitride cantilevers with a resonance frequency of about 300 kHz and a spring constant of about 0.8 N/m (Bruker PFQNE-AL). Silver paste was used to electrically connect the sample with a conductive disc. The topography and contact potential difference (CPD) images (5 × 5 μm^2^, 512 × 512 pixels) were collected at a scan rate of 0.2 Hz. The Fermi level of Ag_2_Se CQDs was obtained from the CPD images using the following equation:  = *E*_F_^tip^ + *e*.CPD; where *e* is the charge of an electron and *E*_F_^tip^ is the work function of the tip. *E*_F_^tip^(= 4.68 eV) was measured by performing FM-KPFM on a gold calibration sample (Bruker PFKPFM-SMPL, *E*_F_^gold^ = 5.1 eV).^61,62^ The average CPD value of gold was obtained from a cross-section line profile of 30-pixel thickness across the reference sample, and that of Ag_2_Se was obtained from the entire scanned sample area.

### 2.9 X-ray Photoelectron Spectroscopy (XPS)

XPS spectra for examining the Ag 3d edge were performed using a Physical Electronics Versaprobe II XPS. Samples were prepared by drop casting 50 μL of Ag_2_Se CQD dispersions in hexane (28 mg/mL) onto glass substrates. For doped samples, 50 μL 1 mg/mL F4-TCNQ in IPA was drop cast onto the center of the CQD film once dry. Spectra were collected using an Al Kα source set to 39.2 W with a 200 μm beam diameter. The survey pass energy was set to 117.40 eV, while the elemental pass energies were set to 23.50 eV. Prior to analysis, spectra were corrected by shifting the C 1s peak to 284.8 eV.

## 3 Results and Discussion

**Figure 1:**
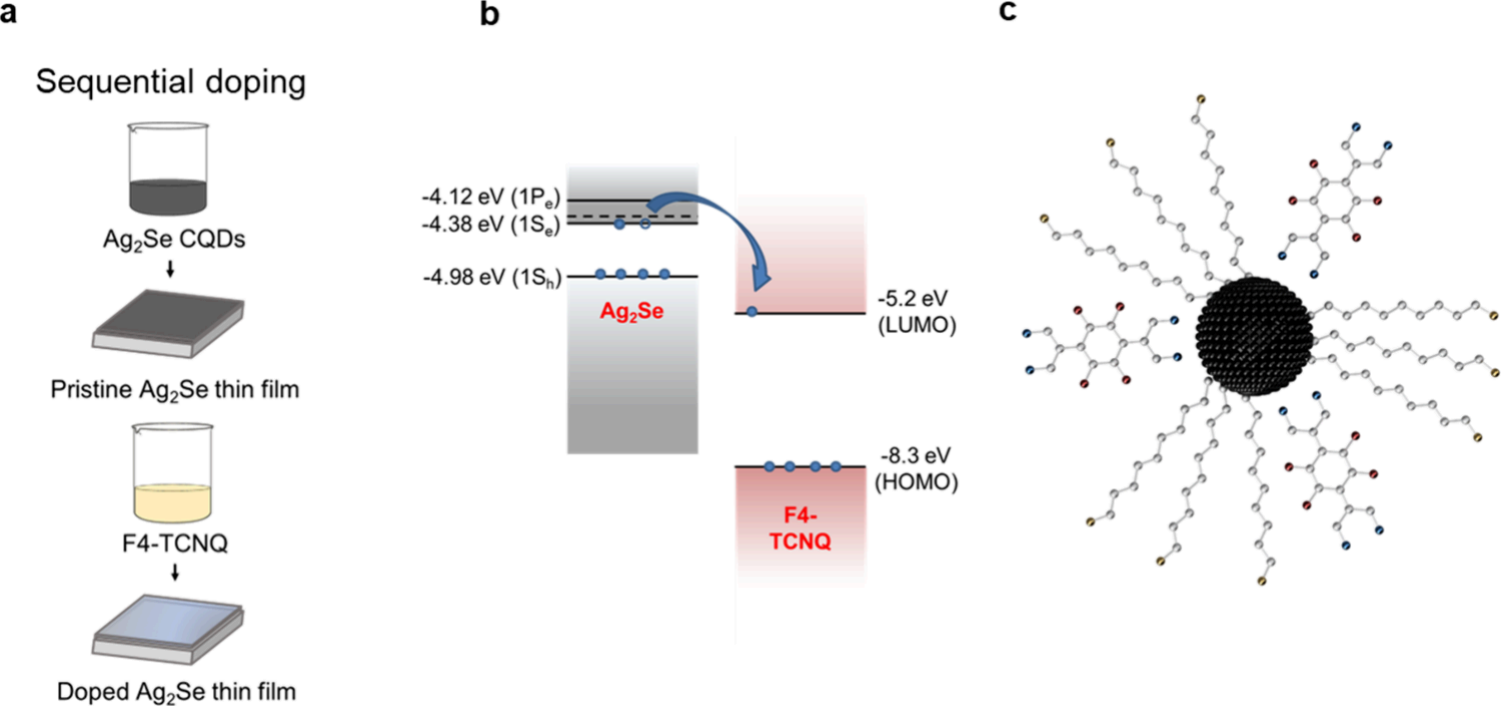
Doping scheme and band edge energy alignment. (a) Sequential doping of the Ag_2_Se CQD thin-film by F4-TCNQ. (b) Type II band edge energy alignment for ∼5 nm Ag_2_Se CQDs^21^ and F4-TCNQ.^45^ The dotted line represents the Fermi level, *E*_F_. (c) Cartoon illustrating a Ag_2_Se CQD with long chain ligands, as well as the hypothesized position of the F4-TCNQ dopants.

NIR and MWIR active Ag_2_Se CQDs were synthesized as described in the Experimental Section based on adapted procedures from previous reports.^13,23^ Briefly, for NIR CQDs, TOP-Se was injected rapidly into a degassed Ag*DDT precursor at 170 °C and grown for 60 min. For MWIR CQDs, TOP-Ag was injected rapidly into a degassed OlAm/TOPO/TOP-Se mixture at 170 °C and the Ag_2_Se CQDs were grown at 160 °C for 12 min. Upon quenching and cleaning, the CQDs were redispersed at 30 mg/mL in a 9:1 hexane:octane solvent mixture for thin-film fabrication through drop casting onto CaF_2_ substrates. The effect of sequential F4-TCNQ doping of as-synthesized (DDT or TOP/OlAm ligands) and ligand exchanged (EDT ligands) Ag_2_Se CQD thin-films was investigated in air-free and in ambient conditions. Figure 1a illustrates sequential doping of Ag_2_Se CQD thin-films with F4-TCNQ, a process where thin-films are fabricated first, followed by dripping of dopant solution onto the film or by soaking the thin-film into the dopant solution. Alternatively, solution doping or vapor doping can be utilized.^63^

For successful coupling and subsequent charge transfer, the dopant (F4-TCNQ) must have an absolute work function (WF) higher than that of the host material (Ag_2_Se) as well as empty states, as demonstrated for ∼5 nm intraband Ag_2_Se CQDs with F4-TCNQ in Figure 1b). Assuming this configuration, based on band edge energies reported in literature,^21,45^ and *E*_F_ confirmed through KPFM studies shown in Section 3 of the SI, electrons from the 1S_e_ (and 1S_h_) state can diffuse to the LUMO level of F4-TCNQ, potentially enabling the ∼0.6 eV interband energy transition ideal for SWIR absorption around ∼2 μm.^22^ Additionally, based on the reported electron affinity (−4.7 eV) of the F4-TCNQ^–^ monoanion,^64^ there is a theoretical possibility of a second electron diffusing from the 1S_e_ state of MWIR Ag_2_Se to the LUMO level of F4-TCNQ^–^, forming the F4-TCNQ^2–^ dianion.

### 3.1 Structural Characterization

**Figure 2:**
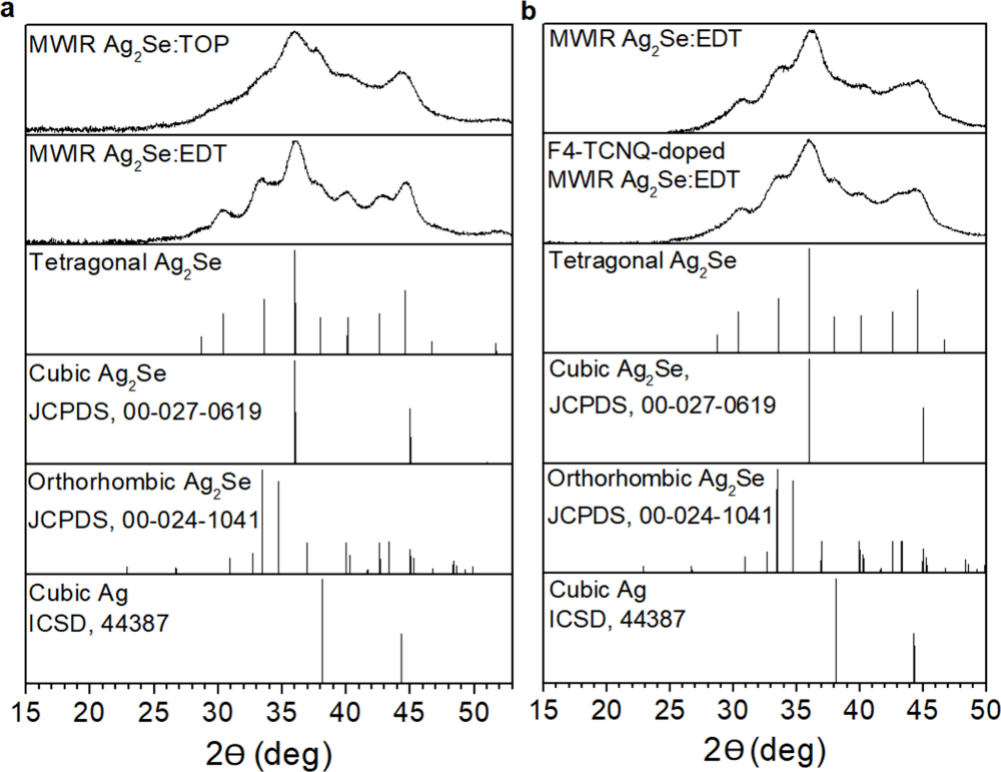
X-ray diffractograms for as-synthesized (MWIR Ag_2_Se:TOP), ligand exchanged (MWIR Ag_2_Se:EDT) and F4-TCNQ doped (F4-TCNQ doped Ag_2_Se:EDT) Ag_2_Se CQDs compared to reference diffractograms for orthorhombic (JCPDS, 00-24-1041), cubic (JCPDS, 00-027-0619) and tetragonal Ag_2_Se^15^ as well as cubic metallic silver (ICSD, 44387). A good match with tetragonal Ag_2_Se is observed. (a) Effect of ligand exchange. Sharpening of peaks after ligand exchange, indicating slight agglomeration during the ligand exchange process. (b) Effect of F4-TCNQ doping. The tetragonal structure is retained.

Figure 2 shows the X-ray diffractogram of as-synthesized (MWIR Ag_2_Se:TOP/OlAm), ligand exchanged (MWIR Ag_2_Se:EDT), and F4-TCNQ doped (F4-TCNQ-doped MWIR Ag_2_Se:EDT) Ag_2_Se CQDs compared to reference diffractograms for tetragonal, cubic (α-Ag_2_Se, JCPDS 00-027-0619), and orthorhombic (α-Ag_2_Se, JCPDS 00-024-1041) Ag_2_Se, as well as cubic elemental silver (Ag^0^, ICSD 44387). It can be observed that the diffractograms of as-synthesized CQDs (Figure 2a) match well with those of cubic and tetragonal Ag_2_Se. After ligand exchange, the diffractograms show an improved match with tetragonal Ag_2_Se and the peaks are slightly sharper, indicating particle growth, likely due to some agglomeration during the ligand exchange. This correlates well with what has been observed in the literature, where as-synthesized Ag_2_Se CQDs exist in a metastable tetragonal phase at room temperature with a crossover size to orthorhombic phase at ∼40 nm.^15,23,65^ Cubic Ag_2_Se overlaps with the tetragonal phase, but is a high-temperature phase, typically the result of a reversible first-order phase transition around ∼135 °C in bulk,^66,67^ and 76–165 °C for nanocrystals depending on size and pressure/shelling.^15^ Therefore, our as-synthesized CQDs are also expected to be tetragonal.

Molecular doping of organic semiconductors typically requires high doping levels (35–50%).^60^ For CQDs, such high doping levels introduce a risk of structural changes to the materials. Figure 2b) shows that the tetragonal crystal structure of MWIR Ag_2_Se:EDT CQDs is retained after F4-TCNQ doping. Thus, we can safely conclude for all further spectroscopic studies that modifications in the crystal structure are not responsible for the observed changes through doping.

### 3.2 Optical Characterization

Optical absorbance can be utilized to monitor the doping of the Ag_2_Se CQD thin films by F4-TCNQ through quenching or shifting of Ag_2_Se peaks, as well as the appearance of absorbance peaks characteristic of different F4-TCNQ species. This could provide clues as to which doping mechanism is dominating. Based on organic semiconductor literature, two main doping mechanisms have been identified for the doping of semiconductors by F4-TCNQ:^42,59^ ground-state charge-transfer complex formation (CPX) and ground-state integer charge transfer (ICT), the latter sometimes defined as ion pair formation (IPA). ICT/IPA results in the formation of an F4-TCNQ^–^ monoanion and a semiconductor cation, analogous to an ionic bond, due to electron transfer from the highest occupied molecular orbital (HOMO)/VB of the (organic) semiconductor to the lowest unoccupied molecular orbital (LUMO) of F4-TCNQ. The formation of a dianion, F4-TCNQ^2–^, is also a possibility.^54,64,68^ Alternatively, through CPX, the frontier orbitals of the semiconductor and the dopant can form a new set of hybridized bonding and antibonding orbitals analogous to those of a covalent bond. In this case, overall charge neutrality is maintained initially, as both electrons from the donor reside in the new hybridized bonding orbital. Then, in a separate step, CPX ionization could take place, forming mobile charges in the material.

The F4-TCNQ^–^ monoanion has characteristic absorbance peaks in the visible range (∼3 eV/413 nm), NIR range (1.43 eV/869 nm and 1.62 eV/767 nm), as well as a characteristic nitrile-stretch in the MWIR range (∼2190 cm^–1^/0.272 eV/4566 nm), which could be observed utilizing UV–vis/FTIR spectroscopy and would be indicative of the ICT mechanism.^60^ The F4-TCNQ^2–^ dianion has no peaks in the NIR, but a broad absorbance feature in the near-ultraviolet (UV) range (∼3.75 eV/330 nm), as well as characteristic nitrile stretches in the MWIR range (∼2167 cm^–1^/0.269 eV/4615 nm and ∼2133 cm^–1^/0.264 eV/4688 nm).^69^ For comparison, charge-neutral F4-TCNQ has an absorbance feature in the visible range (∼3.1 eV/400 nm), as well as a characteristic nitrile-stretch in the MWIR range (2224 cm^–1^/0.276 eV/4496 nm).^60^ The CPX mechanism would be characterized by the lack of anion peaks and the appearance of sub-band gap states, as both the bonding and antibonding orbital of the new hybrid orbital would lie within the band gap of the semiconductor. The appearance of any of these absorbance features after doping could provide indications of a dominating doping mechanism.

#### 3.2.1 Dedoping of NIR Ag_2_Se CQDs

**Figure 3:**
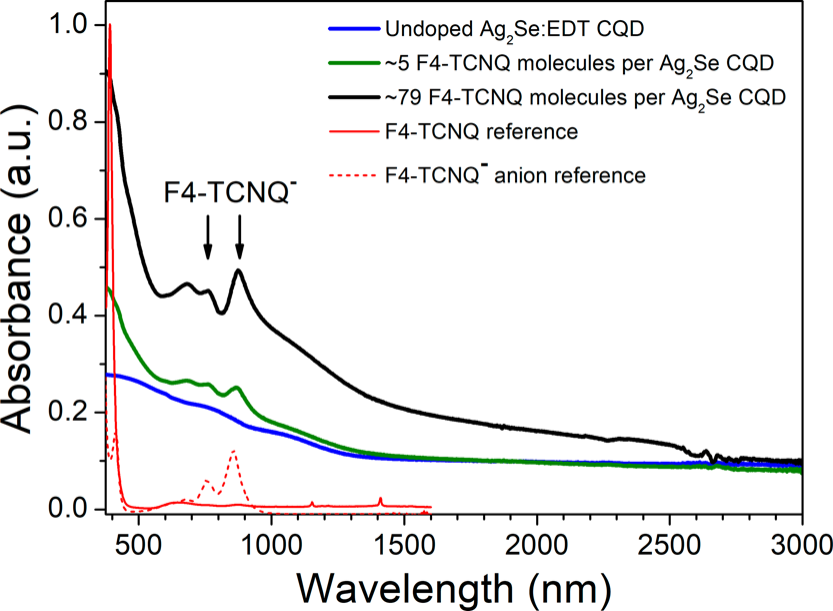
Absorbance spectra for NIR Ag_2_Se:EDT CQDs before (blue solid line) and after exposure to 1 mg/mL F4-TCNQ in chloroform in ambient. Doping concentrations were estimated based on SEM-EDS measurements (Green solid line: 5 ± 3 F4-TCNQ molecules per Ag_2_Se CQD. Black solid line: 79 ± 48 F4-TCNQ molecules per Ag_2_Se CQD). Red lines represent the absorbance spectra for reference samples (Solid: charge-neutral F4-TCNQ. Dashed: F4-TCNQ^–^ anion). The appearance of F4-TCNQ^–^ monoanion peaks is an indication of the ICT doping mechanism, while the appearance of charge-neutral F4-TCNQ peaks indicates excess F4-TCNQ.

Figure 3 shows the optical absorbance in the range 375–3000 nm for a NIR Ag_2_Se:EDT thin-film before and after exposure to 1 mg/mL (0.0036M) F4-TCNQ solution in chloroform. The absorbance spectrum for the ligand-exchanged quantum dot film (blue solid curve) has an onset of absorbance at ∼1350 nm and a second absorbance feature at ∼750 nm. Several new peaks can be observed in the absorbance spectra after the dropwise addition of the F4-TCNQ solution (green and black curves). These can be identified as the F4-TCNQ^–^ monoanion (767/869 nm) and charge-neutral F4-TCNQ (∼400 nm/∼650 nm) peaks through comparison with the dashed and solid red reference spectra for F4-TCNQ^–^ and F4-TCNQ, respectively. The onset of absorption from the Ag_2_Se CQDs around 1300–1400 nm can still be observed after doping. The presence of the monoanion peaks indicates charge transfer from the CQDs to the F4-TCNQ molecules in accordance with the ICT/IPA mechanism,^42^ while the charge-neutral F4-TCNQ features indicate excess F4-TCNQ. The observed absolute dopant concentrations were around ∼0.8% (green curve) and ∼13% (black curve), based on SEM-EDS measurements detailed in Section 2 of the SI, which can be further estimated to be around ∼5 (green curve) and ∼79 (black curve) F4-TCNQ molecules per Ag_2_Se CQD, respectively, confirming excess dopant level.

Zhao et al.^54^ investigated doping of NIR PbS CQDs by F4-TCNQ and observed a reversible blue-shift due to orbital mixing between the VB of the PbS CQDs and the LUMO of F4-TCNQ. In the PbS system, integer electron transfer was not expected due to the unfavorable alignment of band edge energy levels (Δ*E* of −0.23 eV, determined through cyclic voltammetry), although fractional charge transfer signatures were observed through F4-TCNQ^–^ monoanion peaks in the NIR/MWIR range as well as fractional charge transfer products. As observed from Figure S.5 in the SI, the band edge energy alignment between F4-TCNQ and NIR Ag_2_Se CQDs appears favorable for electron transfer, having a Δ*E* value of 0.3 eV.

#### 3.2.2 Dedoping of MWIR Ag_2_Se CQDs

While scientifically interesting, electron extraction from NIR Ag_2_Se CQDs and the potential blue shift of absorbance features might be of less technical importance since many efficient absorbers available for the visible/NIR range already exist.^70^ Dedoping of MWIR Ag_2_Se CQDs, however, could enable a pathway to a viable nontoxic SWIR absorber, which would be of huge importance, as the current state-of-the-art semiconductors in this range are composed of toxic elements, degrade in ambient and/or require cooling during operation.^71,72^

**Figure 4:**
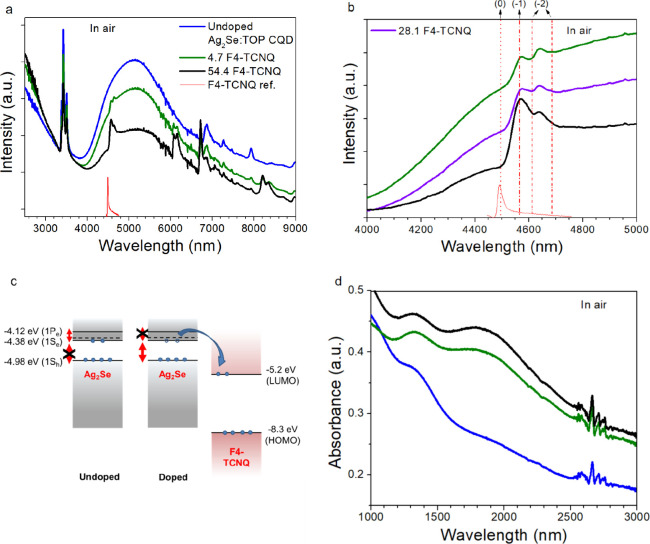
Absorbance spectra for MWIR Ag_2_Se:TOP CQDs before and after exposure to 1 mg/mL of F4-TCNQ in IPA under ambient conditions. Blue curves: Undoped Ag_2_Se:TOP CQDs. Green curves: CQDs with a doping level of 4.7 ± 3.1 F4-TCNQ molecules per Ag_2_Se CQD. Magenta curves: CQDs with a doping level of 28.1 ± 14.0 F4-TCNQ molecules per Ag_2_Se CQD. Black curves: CQDs with doping level 54.4 ± 33.1 F4-TCNQ molecules per Ag_2_Se CQD. Solid and dotted red curves illustrate reference spectra for F4-TCNQ and F4-TCNQ^–^ monoanion, respectively. (a) Extended SWIR/MWIR into the LWIR range. Curves have been shifted in *y*-direction to better visualize the magnitude of the quenching. Peaks at ∼3500 nm are due to C–H stretching in ligands. (b) Close-up of F4-TCNQ nitrile stretch in the air showing initial slight red-shift of nitrile peak from ∼4500 to ∼4565 nm and an extra peak at ∼4650 nm. Vertical lines indicate the expected wavelengths for the nitrile stretch peak(s) for neutral (0, dotted), monoanionic (−1, dash dot), and dianionic (−2, short dash dot) F4-TCNQ. (c) Cartoon illustrating available energy gaps before/after doping of MWIR Ag_2_Se by F4-TCNQ. (d) Area of interest (SWIR) highlighting feature appearing at ∼1900 nm with increasing doping.

**Figure 5:**
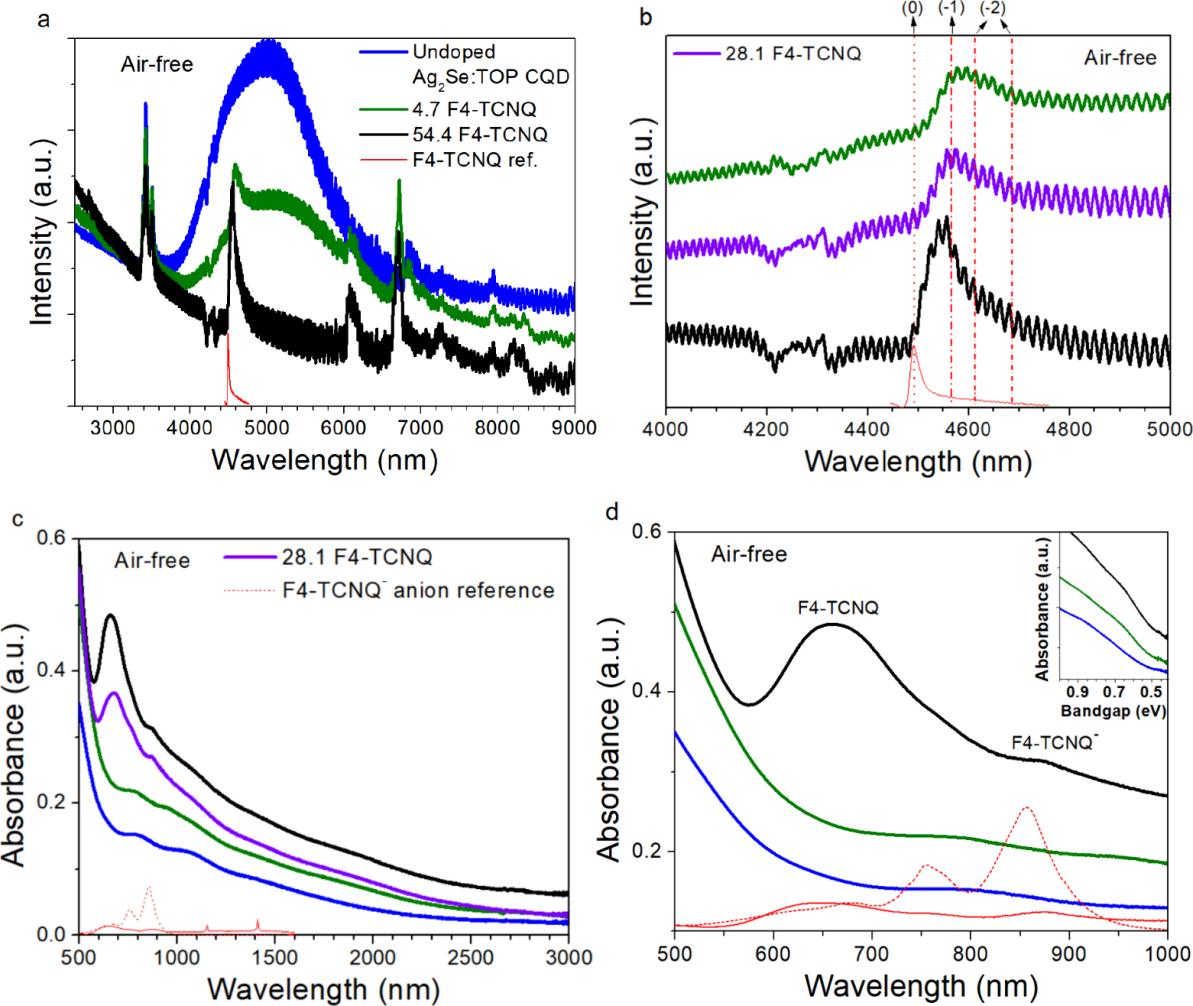
Absorbance spectra for MWIR Ag_2_Se:TOP CQDs before and after exposure to 1 mg/mL of F4-TCNQ in IPA under air-free conditions. Blue curves: Undoped Ag_2_Se:TOP CQDs. Green curves: CQDs with a doping level of 4.7 ± 3.1 F4-TCNQ molecules per Ag_2_Se CQD. Magenta curves: CQDs with a doping level of 28.1 ± 14.0 F4-TCNQ molecules per Ag_2_Se CQD. Black curves: CQDs with doping level 54.4 ± 33.1 F4-TCNQ molecules per Ag_2_Se CQD. Solid and dotted red curves illustrate reference spectra for F4-TCNQ and F4-TCNQ^–^ monoanion, respectively. (a) Extended SWIR/MWIR into LWIR. Curves have been shifted in the *y*-direction to better visualize the magnitude of the quenching. Peaks at ∼3500 nm are due to C–H stretching in ligands. (b) Close-up of air-free F4-TCNQ nitrile stretch showing initial red-shift of nitrile peak from ∼4500 to ∼4600 nm. Curves have been shifted in the *y*-direction to better visualize the red shift. Vertical lines indicate the nitrile stretch peak would be for neutral (0, dotted), monoanionic (−1, dash dot), and dianionic (−2, short dash dot) F4-TCNQ. (c) Visible/NIR/SWIR. (d) Area of interest around characteristic NIR monoanion peaks. Inset: Highlighting feature appearing around ∼0.65 eV/∼1900 nm with increasing doping.

Figures 4 and 5 show absorbance spectra in ambient and air-free conditions, respectively, for as-synthesized MWIR Ag_2_Se CQD thin films before and after exposure to 1 mg/mL (0.0036M) F4-TCNQ in IPA. The switch to a more polar solvent for F4-TCNQ was due to the partial dissolution of the CQD thin films exposed to chloroform, as illustrated in Figures S.6 and S.7 in the SI. The comparison of F4-TCNQ doping both in air-free and ambient conditions was triggered by observations of loss of doping in ambient for other molecular dopant systems for CQDs.^73^

From Figure 4a, presenting absorbance in the range 2500–9000 nm under ambient conditions, a strong MWIR peak at ∼5100 nm can be observed prior to F4-TCNQ exposure (blue solid curve), which is partially quenched upon dropwise exposure to 0.0036 M F4-TCNQ in IPA (green and black solid curves). The quenching of the MWIR peak indicates charge transfer, likely from the 1S_e_ state of the Ag_2_Se CQD to the LUMO of F4-TCNQ, as illustrated in Figure 4c. The blocking of the intraband gap and the corresponding increased interband absorbance at ∼2500 nm as a result of increasing F4-TCNQ doping is a promising indicator for achieving SWIR activity through the doping process. Alternatively, MWIR peak quenching could indicate particle aggregation; however, this can be ruled out based on the XRD result in Figure 2b, demonstrating that the tetragonal structure is retained after doping.

Figure 4b shows the shift of the nitrile stretch signature of the F4-TCNQ molecule due to the interaction with MWIR Ag_2_Se CQDs. It is known in literature that the negative charge in the F4-TCNQ^–^ monoanion resides on the nitrile bond resulting in a red-shift (∼33 cm^–1^) in the vibrational spectrum compared to the neutral bond.^60^ Even at the lowest F4-TCNQ dopant concentration (4.7 F4-TCNQ molecules per Ag_2_Se CQD, 0.8% absolute F4-TCNQ concentration), the nitrile peak red-shifts to ∼4565 nm, indicating the presence of the F4-TCNQ^–^ monoanion. These observations indicate that there is a charge transfer between the MWIR Ag_2_Se CQDs and the F4-TCNQ and that the doping mechanism is ICT.^60^ An additional peak at ∼4650 nm also appears after doping, likely the signature of other known vibrational modes of F4-TCNQ or F4-TCNQ^–^,^64,74^ fractional charge transfer forms of F4TCNQ,^54^ or potentially oxidized F4-TCNQ species.

In Figure 4d, showing the range 1000–3000 nm, a new, broad peak around ∼1900 nm is observed after doping, matching the predicted interband gap for these CQDs based on the two-band k.p model discussed in Section 1 in the SI,^23,38^ indicating enabling of the interband gap with F4-TCNQ doping. The broadness of this peak is likely a result of slight differences in the absolute interband energy gap due to the size distribution of the CQDs, or due to the dopant concentration distribution on the CQD population. In the visible – NIR range, shown in Figure S.8b,d, only faint and broad features in the range where the characteristic NIR monoanion peaks are expected can be observed, owing to overlapping absorption from the SWIR absorbing Ag_2_Se CQDs.

A twin set of samples was exposed to a similar doping procedure as in Figure 4 except under air-free conditions, with results visualized in Figure 5. Under air-free conditions, more complete quenching of the MWIR peak around ∼5000 nm by F4-TCNQ doping can be observed (Figure 5a), coupled with increased absorbance at ∼2500 nm. More complete quenching under air-free conditions could be due to the absence of an environmental effect, which under ambient conditions might replenish the doped CQDs with electrons causing only partial quenching. The red shift of the nitrile stretch originally at ∼4500 nm, shown in Figure 5b, is similar under air-free conditions and in air, suggesting a high degree of charge transfer. However, under air-free conditions, the nitrile stretch peak gradually blue-shifts back toward ∼4500 nm with the addition of F4-TCNQ in IPA, indicating an increased concentration of charge-neutral F4-TCNQ based on comparison with F4-TCNQ reference spectra (red solid curve).

From Figure 5c, presenting air-free absorbance data in the range 500–3000 nm, as well as a close up of the range 500–1000 nm in Figure 5d, it can be observed that for ∼28.1 F4-TCNQ molecules per Ag_2_Se CQD (4.7% absolute F4-TCNQ concentration, magenta curve) and ∼54.4 F4-TCNQ per Ag2Se CQD (9.2% absolute F4-TCNQ concentration, black curve), the characteristic absorbance features of the F4-TCNQ^–^ monoanion and charge neutral F4-TCNQ appear and grow in intensity. A growing peak characteristic of the charge-neutral F4-TCNQ peak around ∼400 nm can also be observed in Figure S.8a in the SI. This strengthens the hypothesis that doping occurs through the ICT mechanism with an excess of charge-neutral F4-TCNQ.^60^

At 4.7 F4-TCNQ molecules per Ag_2_Se CQD (0.8% absolute F4-TCNQ concentration, green curve), no characteristic F4-TCNQ or F4-TCNQ^–^ features can be observed in the visible and NIR range, although quenching of the MWIR peak is observed in Figure 5a indicating charge transfer. In Figure S.8c, the near UV peak of the F4-TCNQ^2–^ dianion can be observed at 4.7 F4-TCNQ but is masked by the growing charge-neutral F4-TCNQ peak at higher concentrations. A good match with the two MWIR peaks (at 4615 and 4688 nm) characteristic of the dianion is not observed in Figure 5b. Dominating dianion formation at low F4-TCNQ dopant concentrations has been reported previously in organic semiconductors, limited by the physical space available to accommodate two polarons.^64^

A faint absorbance feature at ∼1900 nm can be observed at the highest dopant concentration (54.4 F4-TCNQ, black solid curve) in Figure 5c and the inset of Figure 5d, which is close to the expected interband gap (∼2000 nm) for these CQDs. However, under air-free conditions, the peak around ∼1900 nm is much less prominent than under ambient conditions, which we hypothesize might be due to the extraction of electrons not only from the S_e_ state but also from the 1S_h_ state under air-free conditions where replenishment of electrons by the environment is excluded. The excess doping would thus lead to a reduced band edge absorption at ∼1900 nm.

Koh et al.^73^ indicated that molecular (cobaltocene) doping of (PbS) CQDs can be unstable under ambient conditions. To test this for our system, the MWIR Ag_2_Se CQD film exposed to F4-TCNQ under air-free conditions was exposed briefly to air, and the absorbance spectra were remeasured. As shown in Figure S.9 in the SI, the brief exposure to air did not significantly change the absorbance spectra of F4-TCNQ doped Ag_2_Se CQDs. This could indicate that the 1S_e_ state of the Ag_2_Se^+^-F4-TCNQ^–^ complex is higher in energy than the *E*_F,env_, however, this would have to be confirmed via ultraviolet photoelectron spectroscopy (UPS) or (spectro)electrochemistry measurements.^36^

The similarities in the absorbance spectra under air-free conditions and in ambient–quenching of the MWIR peak, (faint) monoanion peaks in the NIR range, shifting of the F4-TCNQ nitrile stretch in the MWIR range, and increased absorbance in the SWIR range–lead us to conclude that a significant degree of charge transfer, δ, is taking place between F4-TCNQ and MWIR-active Ag_2_Se:TOP CQDs. δ can be calculated based on the observed frequency shift, Δν, of the nitrile stretch through eq 1,^60^ which can help shed some light on the dominating doping mechanism:
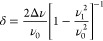
1ν_0_ and ν_1_ are the vibrational frequencies of the neutral F4-TCNQ dopant (2227 cm^–1^) and the radical monoanion (2194 cm^–1^), respectively.^60,74^ Based on the large shift (Δν = 40–43 cm^–1^) observed when comparing the location of the nitrile peak at the lowest dopant concentration (green curves in Figures 4b and 5b) with the reference spectra for F4-TCNQ (red curve), a δ of ∼1 can be calculated both under ambient and air-free conditions, indicating significant charge transfer. These high degrees of charge transfer strengthen our hypothesis of ICT as the dominant mechanism in this system. With such strong evidence of charge transfer in the MWIR range, the weak monoanion features in the NIR range can thus be explained through masking by the charge-neutral F4-TCNQ peaks due to significant F4-TCNQ excess (F4-TCNQ molecule to Ag_2_Se CQD ratios up to ∼54), as seen through the strong peaks at 600 and 400 nm in Figures 5d and S.8 in the SI. Yet, corresponding to a maximum absolute doping level of ∼9%, more efficient doping is achieved for Ag_2_Se CQDs than in organic semiconductors (requiring 35–50% doping levels).^60^

Additional absorbance spectra for MWIR-active Ag_2_Se CQD thin-films are shown in Figures S.10–S.15 in Section 4 of the SI, illustrating the doping effect on ligand exchanged (EDT) thin-films, utilizing alternate solvents for F4-TCNQ, varying F4-TCNQ concentration, and F4-TCNQ doping through soaking instead of dripping. In all cases, quenching of the MWIR peak was observed; however, the interband gap feature at ∼1900 nm was only observed with the original long-chain ligands, as shown in Figures 4 and 5. Soaking the film in the same solution as used for dripping did not yield the same absorbance response. It was also observed that the F4-TCNQ concentration and choice of solvent seem to play an important role. F4-TCNQ has a relatively low solubility in many solvents,^75,76^ and from the organic semiconductor literature, it has been shown that the choice of solvent can affect the doping mechanism.^77^

It should be noted that the doping process is likely significantly more complex than the limited parameter space considered above. A recent report indicates that, in addition to the relative positions of band edge energies–electrostatic interactions, the size and bulkiness of the molecules and formed complexes as well as the absolute doping level play roles in determining the doping mechanism.^64^ The complex interplay of F4-TCNQ, Ag_2_Se CQDs, ligands on the CQD surface, and formed charge transfer complexes therefore calls for a detailed computational investigation in order to obtain a more complete theoretical understanding of the doping process.

### 3.3 X-ray Photoelectron Spectroscopy (XPS)

**Figure 6:**
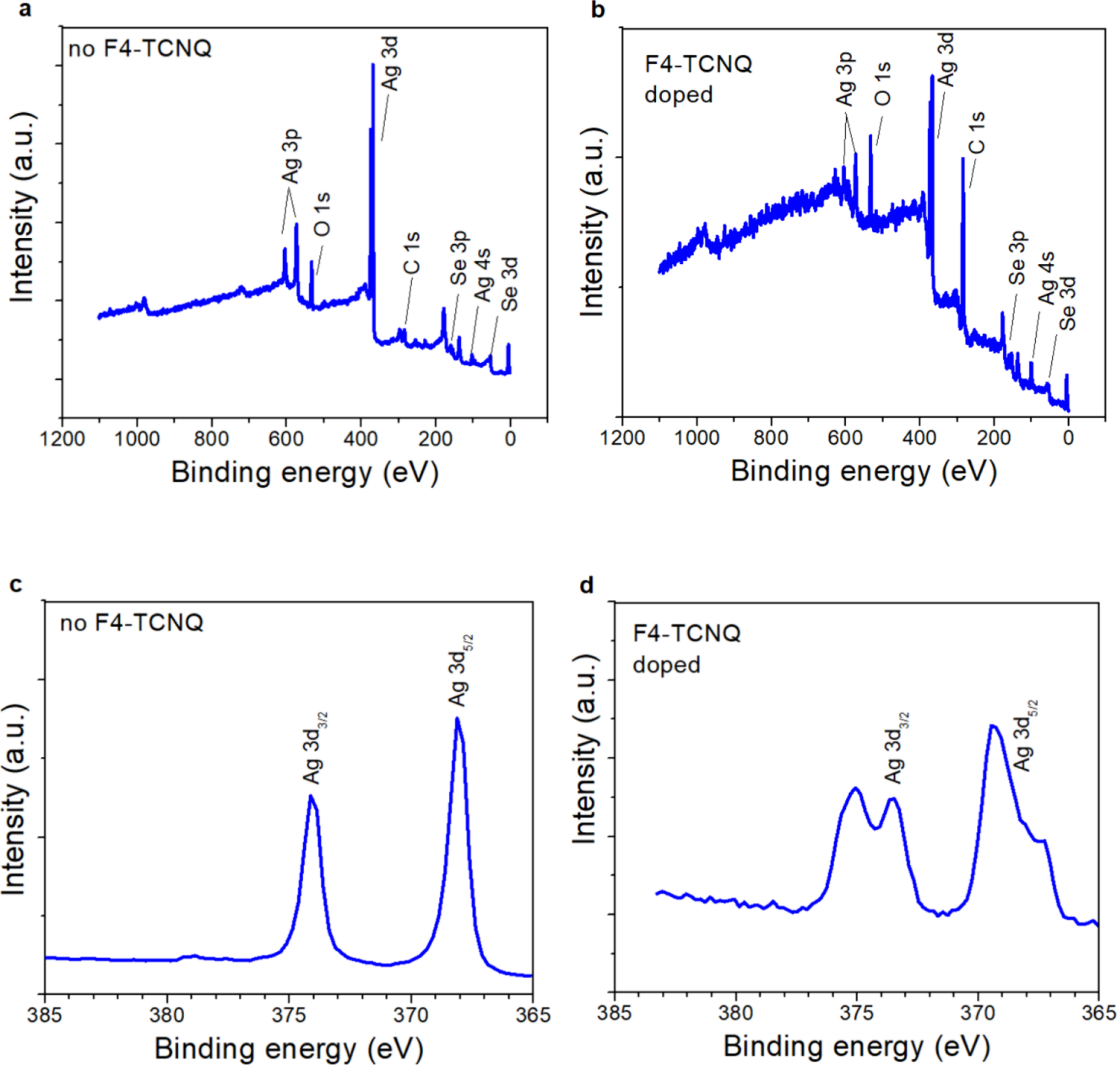
XPS data for undoped and F4-TCNQ doped Ag_2_Se CQD films. (a) Survey scan for undoped film, identifying characteristic peaks of Ag 3p, O 1s, Ag 3d, C 1s, Se 3p, Ag 4s, and Se 3d. (b) Survey scan after F4-TCNQ doping. (c) Ag 3d edge for undoped film. (d) Ag 3d edge after F4-TNCQ doping. Doublet peaks are observed around the characteristic peaks of Ag 3d_3/2_ and Ag 3d_5/2_, possibly indicating the presence of Ag(III) moieties due to oxidation.

XPS analyses of undoped and doped Ag_2_Se CQDs provide additional clues in terms of indicating which species are present close to the surface after exposure to F4-TCNQ. Figure 6 shows the results of an XPS study of Ag_2_Se CQD thin films on glass before and after exposure to F4-TCNQ. The survey scans in Figure 6a,b identify similar groups of characteristic peaks, including Ag, O, C and Se. The scan of the Ag 3d edge in Figure 6c before doping confirms that Ag is present, identified as characteristic singlet peaks of Ag 3d_3/2_ and Ag 3d_5/2_.^14^ After exposure to F4-TCNQ, we observed the formation of satellite peaks at 3d_3/2_ and 3d_5/2_ in the F4-TCNQ doped sample. These satellites are not present in the undoped sample. We attribute this to the formation of oxidized Ag (III) species, which might occur due to the charge transfer between F4-TCNQ and Ag_2_Se. Similar Ag (III) peaks have also been observed in literature.^78,79^

### 3.4 Potential as a Photodetector

The ultimate demonstration of the strategy discussed in this study would be to fabricate a SWIR photodetector based on F4-TCNQ doped MWIR Ag_2_Se CQDs. For efficient photodetection, the CQD absorber must be well passivated and have low trap densities, display limited Auger recombination, and the exciton lifetime must be long enough for the carriers to be transported out of the absorber layer before recombination.^7^ Preliminary photodetector fabrication was attempted; however, the devices were not photoresponsive after F4-TCNQ doping. A possible reason for this could be the trapping of photogenerated carriers by excess F4-TCNQ, as reported by Zhao et al.^54^

Zhao et al.^54^ doped PbS CQDs of various sizes with F4-TCNQ and observed a promising blue shift in absorption. However, PL quenching was also observed, likely due to the photoinduced CQD to F4-TCNQ electron transfer. PL quenching was also observed through TCNQ doping of PbS CQDs.^56^ This could be a showstopper for F4-TCNQ doped Ag_2_Se CQDs as well, as photoexcited carriers would be absorbed by excess F4-TCNQ molecules on the surface of the CQDs instead of being transported to the contacts. Keeping the excess F4-TCNQ to a minimum would therefore be of critical importance; hence, we are devising ways to quench the MWIR peak and enable the SWIR absorbance with controlled amounts of F4-TCNQ. Doping of bulk Ag_2_Se by F4-TCNQ can also be a feasible approach, as demonstrated in Figures S.14 and S.15 in Section 5 of the SI, even with a much lower surface area/volume ratio than nanomaterials.

### 3.5 Alternative Strategies

Alternative dopants reported in the literature for tuning the degree of n-type doping in CQDs are sulfide (S^2–^) and [6,6]-phenyl-C_61_-butyric acid methyl ester (PCBM), which are discussed in Section 6 in the SI.

Another reported method for observing the dedoping of Ag_2_Se CQDs is through spectroelectrochemical studies. By depositing the CQD layer onto a working electrode submerged in an electrolyte and applying a constant electrochemical potential in the range of −0.4 to 0.6 V to reduce/oxidize the nanocrystal, Park et al.^22^ were able to tune the absorption characteristics of the CQD and identify the origin of MWIR electronic transitions. While this technique is great for showing the impact of doping, relying on a liquid electrolyte, it cannot be used in a practical photodetector device.

Alternative strategies for achieving SWIR-active Ag_2_Se CQDs besides direct colloidal synthesis or doping could be to utilize cation exchange protocols based on cadmium selenide (CdSe) or PbSe CQDs.^25,80,81^ Son et al.^80^ demonstrated reversible cation exchange from CdSe CQDs to Ag_2_Se CQDs in solution for initial particle size 4.1 nm, reporting loss and subsequent recovery of absorbance in the range 350–850 nm. Bera et al.^25^ demonstrated self-doped Ag_*x*_Se (*x* > 2) CQDs with a steady-state intraband transition up to ∼3.1 μm, initially starting with CQDs with a diameter of 3.9 nm. It can be hypothesized that starting with even smaller CdSe or PbSe CQDs could yield Ag_2_Se CQDs with absorption in the SWIR. Alternatively, a hybrid approach utilizing cation exchange and remote doping with small molecules could be beneficial to enable the currently inaccessible SWIR region of the electromagnetic spectrum for Ag_2_Se CQDs.

## 4 Conclusions

For Ag_2_Se CQD-based IR photodetectors to compete fully with detectors based on toxic nanocrystalline semiconductors, access to the full range from NIR to MWIR via SWIR must be established. While protocols enabling the synthesis of NIR- and MWIR-active Ag_2_Se CQDs from the same precursor ratios and growth temperature have been demonstrated, direct synthesis of SWIR-active CQDs is currently not available. In general, limited reports exist on SWIR Ag_2_Se CQD photodetectors. We here propose an alternative strategy to the direct synthesis of SWIR-active CQDs. Through dedoping of intraband MWIR-active Ag_2_Se CQDs using the strong electron acceptor F4-TCNQ, we hypothesize that the interband energy gap for absorption of SWIR photons can be accessed through the extraction of electrons from the 1S_e_ state. Through optical spectroscopy studies, we initially demonstrate integer charge transfer between NIR-active Ag_2_Se CQDs and F4-TCNQ, as opposed to an orbital mixing mechanism which has been suggested for doping of NIR PbS CQDs by F4-TCNQ. Moving on to more technologically relevant MWIR-active Ag_2_Se CQDs, we demonstrate quenching of the MWIR Ag_2_Se absorption peak, shifting of nitrile vibrational peaks characteristic of charge-neutral F4-TCNQ, as well as enhanced CQD absorption around ∼2500 nm after doping, while retaining the tetragonal Ag_2_Se crystal structure. Similar indications of integer charge transfer between MWIR Ag_2_Se CQDs and F4-TCNQ are achieved both in ambient and under air-free conditions. These are promising milestones on the path to achieving SWIR Ag_2_Se CQD photodetectors.

## Supporting Information Available

Two-band k.p model; evaluation of doping concentration; determination of Fermi level of Ag_2_Se CQDs; additional optical spectroscopy data for doping of NIR and MWIR Ag_2_Se CQDs by F4-TCNQ; doping of bulk Ag_2_Se by F4-TCNQ; and other doping strategies, including Supporting Figures S1–S17 (PDF)
