# Supplementary material for: Dedoping of Intraband Silver Selenide Colloidal Quantum Dots through Strong Electronic Coupling at Organic/Inorganic Hybrid Interfaces
Source: Cryst Growth Des. 2024 Mar 22;24(7):2821–32. doi: 10.1021/acs.cgd.3c01474 (PMC10995946; doi:10.1021/acs.cgd.3c01474)
Supplement: Supplementary file 1 — cg3c01474_si_001.pdf [file cg3c01474_si_001.pdf]

Supporting Information

De-doping of Intraband Silver Selenide Colloidal Quantum Dots through Strong Electronic Coupling at Organic/Inorganic Hybrid Interfaces

Håvard Mølnås[a], Shlok Joseph Paul[a], Michael R. Scimeca[a], Navkawal Mattu[a], Jiaqi Zuo[a], Nitika Parashar[a], Letian Li[a], Elisa Riedo[a], and Ayaskanta Sahu[a,*]

[a]Department of Chemical and Biomolecular Engineering, Tandon School of Engineering, New York University, Brooklyn, New York 11201, United States of America

*Correspondence to: asahu@nyu.edu

Section 1: Two-band k.p. model

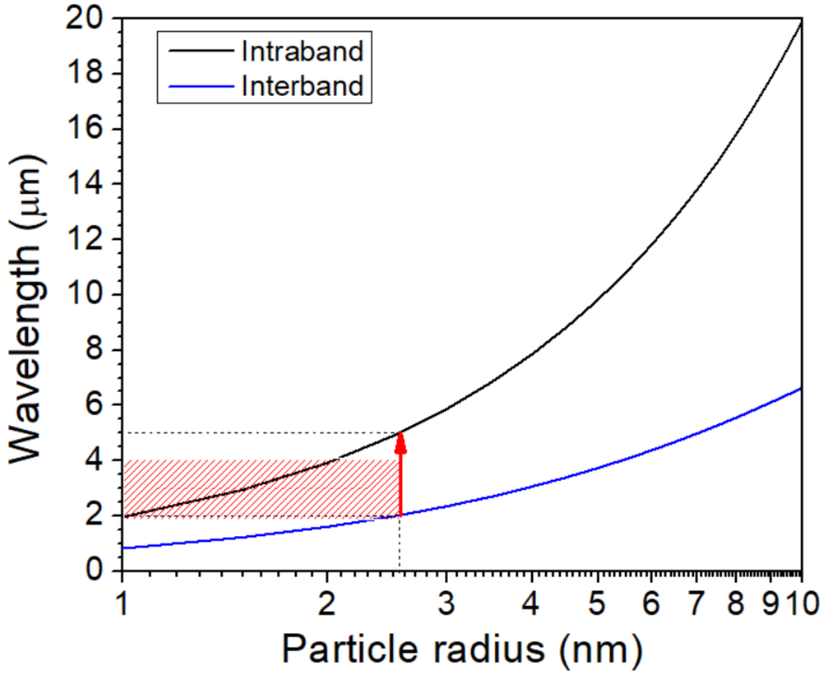

Figure S.1. Calculated intra- and interband transitions VS particle radius for Ag_2Se CQDs based on a two-band k.p model.[1,2] Dotted black lines indicate crossover from interband to intraband at diameter ~5.1 nm. Red shaded area indicates wavelengths (2 – 4 μm) currently inaccessible through direct hot-injection synthesis.

The two-band k.p model originally demonstrated for HgTe CQDs by Lhuillier et al.[1] was recently adapted to Ag_2Se CQDs by Scimeca et al.,[2] shown in **Figure S.1** and expressed mathematically in Eq. S.1 and S.2:

$$E_{inter} = -\frac{E_G}{2} \pm \sqrt{\frac{E_G^2}{4} + \frac{2}{3}E_p\frac{\hbar k_{1S}^2}{2m_0}} \quad \text{(S.1)}$$

$$E_{intra} = \sqrt{\frac{E_G^2}{4} + \frac{2}{3}E_p\frac{\hbar k_{1P}^2}{2m_0}} - \sqrt{\frac{E_G^2}{4} + \frac{2}{3}E_p\frac{\hbar k_{1S}^2}{2m_0}} \quad \text{(S.2)}$$

where E_{inter} is the interband energy gap between the $1S_h$ and $1S_e$ states, E_{intra} is the intraband gap between the $1S_e$ and $1P_e$ states, E_G is the bulk bandgap, E_p is the Kane

parameter, $\hbar$ is the reduced Planck's constant, m_0 is the free electron mass, $k_{1S} = \frac{\pi}{R}$, $k_{1P} = \frac{4.49}{R}$, and R is the radius of the particle. Utilizing a bulk bandgap of 0.07 eV and Kane parameter 8.7 eV,[3] the latter based on fitting of the model to experimental size and absorbance data,[2] the interband gap for Ag_2Se CQDs with a certain intraband gap can be predicted. Specifically, for our MWIR active Ag_2Se CQDs with an intraband energy gap of ~5 µm/0.248 eV, the interband gap would be in the SWIR (~2 µm/0.62 eV).

Section 2: F4-TCNQ concentration in doped CQD films

The elemental ratios were determined based on SEM-EDS measurements using a TM3000 Hitachi tabletop SEM with a Bruker EDS System and QUANTAX 70 software. The Ag_2Se CQD thin-films were drop cast on sapphire substrates, and ligand exchanged and doped with F4-TCNQ in an identical procedure to the samples used for optical characterization, as described above. The absorbance spectra in the range 2500 – 9000 nm were taken in a Thermo Scientific Nicolet iS50 FT-IR spectrometer with Omnic software to ensure comparable development of the spectra to Figure 4a) and Figure 5a).

Using SEM-EDS, elemental ratios were determined at three different spots on the films and averaged for further calculations of dopant concentration. Converting from at% elemental fluorine (F) and Ag to F4-TCNQ/Ag_2Se CQD ratios, the following assumptions were made:

1. The radius of Ag_2Se CQDs with peak intraband absorption at 5.1 µm is 2.6 nm (in accordance with a two-band k.p model).[1,2]
2. The Ag_2Se CQDs exist in a tetragonal crystal structure at room temperature with lattice parameters a=b=0.706 nm and c=0.498 nm.[4]
3. Each F4-TCNQ molecule contains four F atoms.[5]

Based on the above assumptions, the volumes of the tetragonal unit cell and the Ag_2Se CQD can be compared, yielding 297 unit cells per CQD and a total of 1188 Ag atoms per CQD. This enables the calculation of F4-TCNQ/Ag_2Se molecular and CQD ratios for 0, 1, 2, and 3 drops of added 1 mg/mL F4-TCNQ in IPA, as shown in **Figure S.2** and **Figure S.3**, respectively:

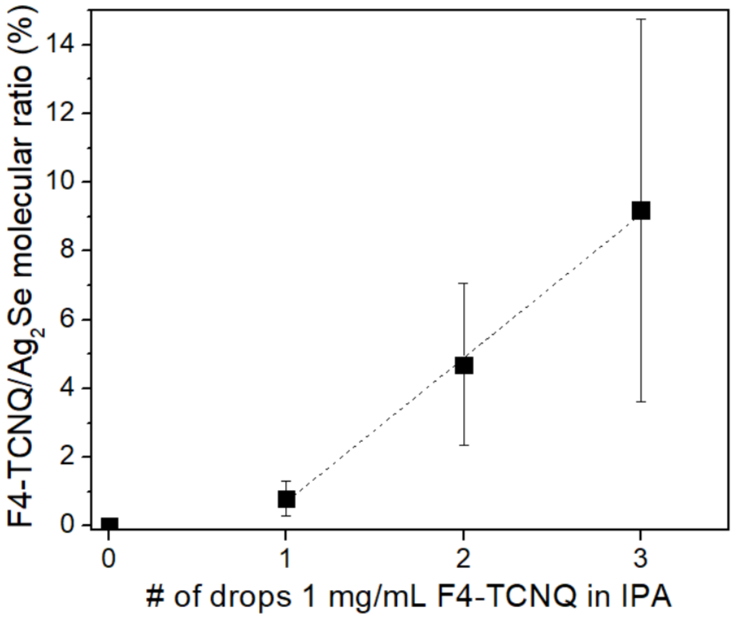

Figure S.2. F4-TCNQ/Ag_2Se molecular ratio for our doping experiments with the addition of 0, 1, 2, and 3 drops of 1 mg/mL F4-TCNQ in IPA.

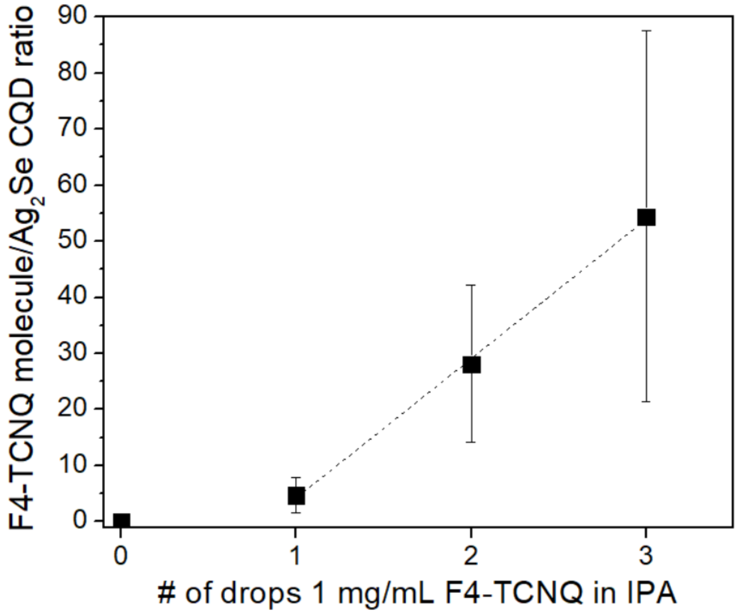

Figure S.3. F4-TCNQ molecule/Ag_2Se CQD ratio for our doping experiments with the addition of 0, 1, 2, and 3 drops of 1 mg/mL F4-TCNQ in IPA.

The data points for 1 - 3 drops of 1 mg/mL F4-TCNQ in IPA in Figure S.3 can be fitted to a linear trend, $y = ax + b$, having a slope of 24.85, allowing for extrapolation.

Section 3: Determination of Fermi level of Ag_2Se CQDs

The Fermi level, E_F, of our NIR Ag_2Se:EDT CQDs was determined to -4.286 eV relative to vacuum based on KPFM measurements, **Figure S.4**. This is a good match with the reported E_F of 5 nm Ag_2Se CQDs (-4.3 eV).[6] Our obtained E_F is also in the middle of the reported band gap of NIR Ag_2Se CQDs,[7] **Figure S.5**, resulting in a theoretical Type II band alignment with respect to F4-TCNQ.

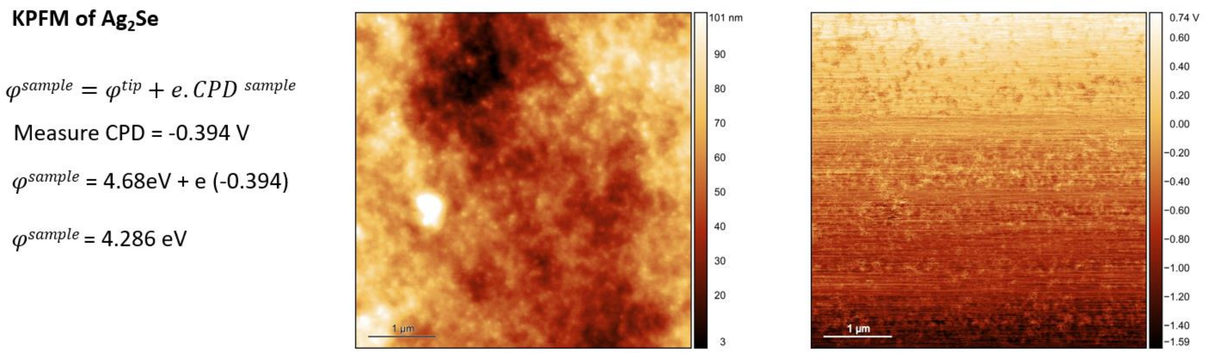

Figure S.4. Work function determination through KPFM of NIR Ag_2Se:EDT CQDs.

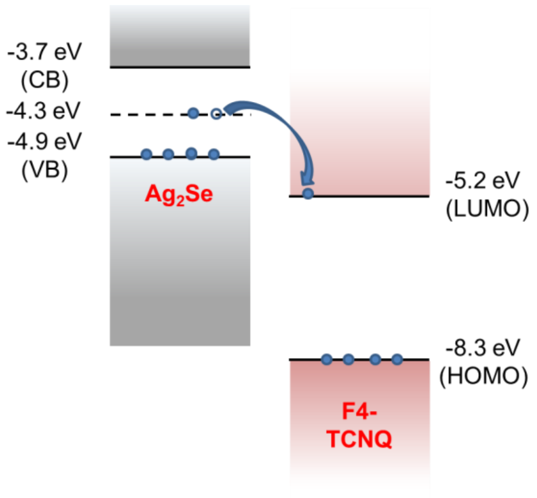

Figure S.5. Type II band edge energy alignment for NIR Ag_2Se CQDs[7] and F4-TCNQ.[8] The dotted line represents the Fermi level, E_F, determined by KPFM, as shown in Figure S.4.

Section 4: Additional optical absorbance data

Figure S.6 shows absorbance spectra for MWIR Ag_2Se CQDs with 1-dodecanethiol (DDT) ligands on a calcium fluoride (CaF_2) window before and after sequential doping with F4-TCNQ dissolved in chloroform (1 mg/mL). Monoanion peak pair formation can be observed around 800 nm, although only at extremely high doping concentrations, causing aggregation of particles. XRD experiments confirmed beginning aggregation and transition to the orthorhombic phase, which has been reported to occur at particle sizes approaching 40 nm.[4] It was hypothesized that the long DDT carbon chain ligands prevented efficient charge transfer until high concentrations of dopants were reached, which then resulted in particle aggregation. Shorter ligands are expected to reduce this issue, thus we employed 1,2-ethanedithiol (EDT) in the following experiments. Additionally, the chloroform solvent diluted the film, as illustrated in **Figure S.7**, thus the need to switch to an orthogonal solvent such as isopropyl alcohol (IPA) or acetone.

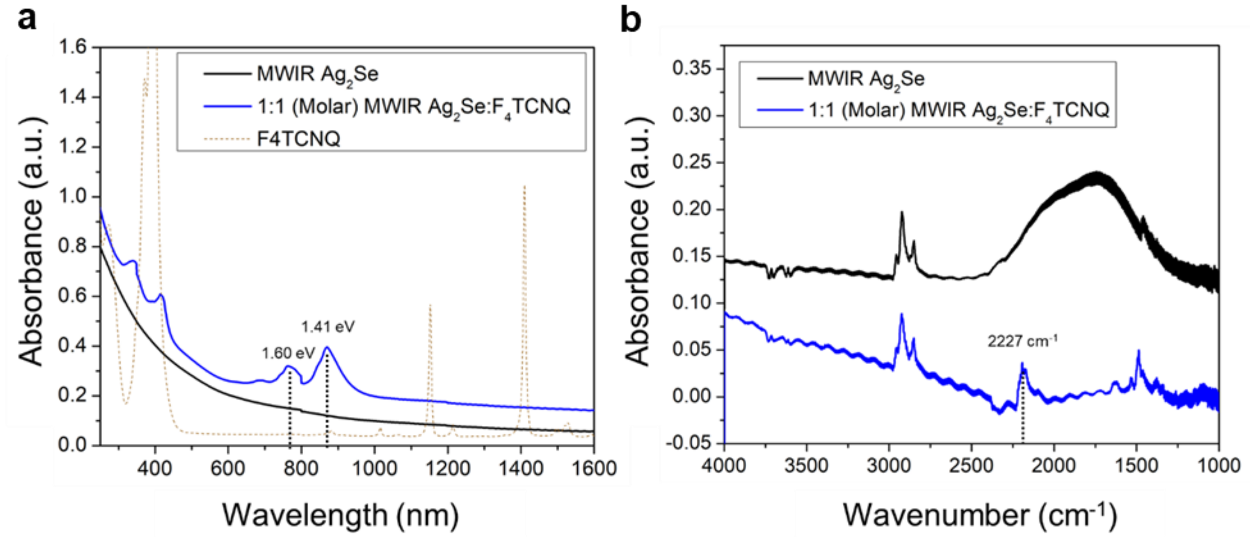

Figure S.6. UV-Vis-NIR (a) and FTIR (b) absorbance spectra of MWIR Ag_2Se:DDT before and after doping with F4-TCNQ in chloroform (1 mg/mL). The addition of F4-TCNQ creates a monoanion radical pair as seen in (a), but only at extremely high concentrations that aggregate the particles.

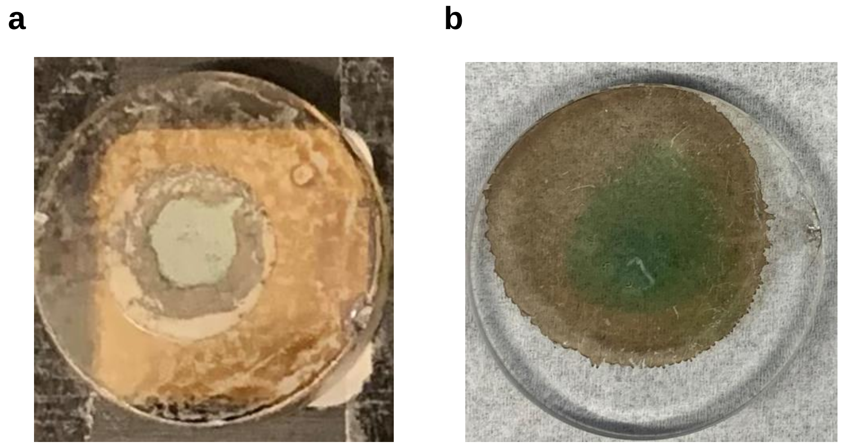

Figure S.7. F4-TCNQ doped MWIR Ag_2Se CQD films on CaF_2 windows through dripping of F4-TCNQ in chloroform (a) and IPA (b). The use of chloroform dissolves/dilutes the Ag_2Se CQD film.

In the remainder of this section, additional absorbance spectra for MWIR-active Ag_2Se CQD thin-films are presented, illustrating the doping effect on ligand exchanged (EDT) thin-films, utilizing alternate solvents for F4-TCNQ, varying F4-TCNQ concentration, and F4-TCNQ doping through soaking instead of dripping. In all cases, quenching of the MWIR peak was observed, however, the interband gap feature at ~1900 nm was only observed with the original long-chain ligands, as shown in Figure 4 and Figure 5 in the main text. Soaking the film in the same solution as used for dripping did not yield the same absorbance response. It was also observed that the F4-TCNQ concentration and choice of solvent seem to play an important role. F4-TCNQ has a relatively low solubility in many solvents,[9,10] and from the organic semiconductor literature, it has been shown that the choice of solvent can affect the doping mechanism.[11]

Figure S.8 shows the full absorbance spectra in the Vis/NIR/SWIR range for MWIR Ag_2Se:TOP CQDs before and after exposure to 1 mg/mL F4-TCNQ in IPA under air-free (Figure S.8a,c) and ambient (Figure S.8b,d) conditions.

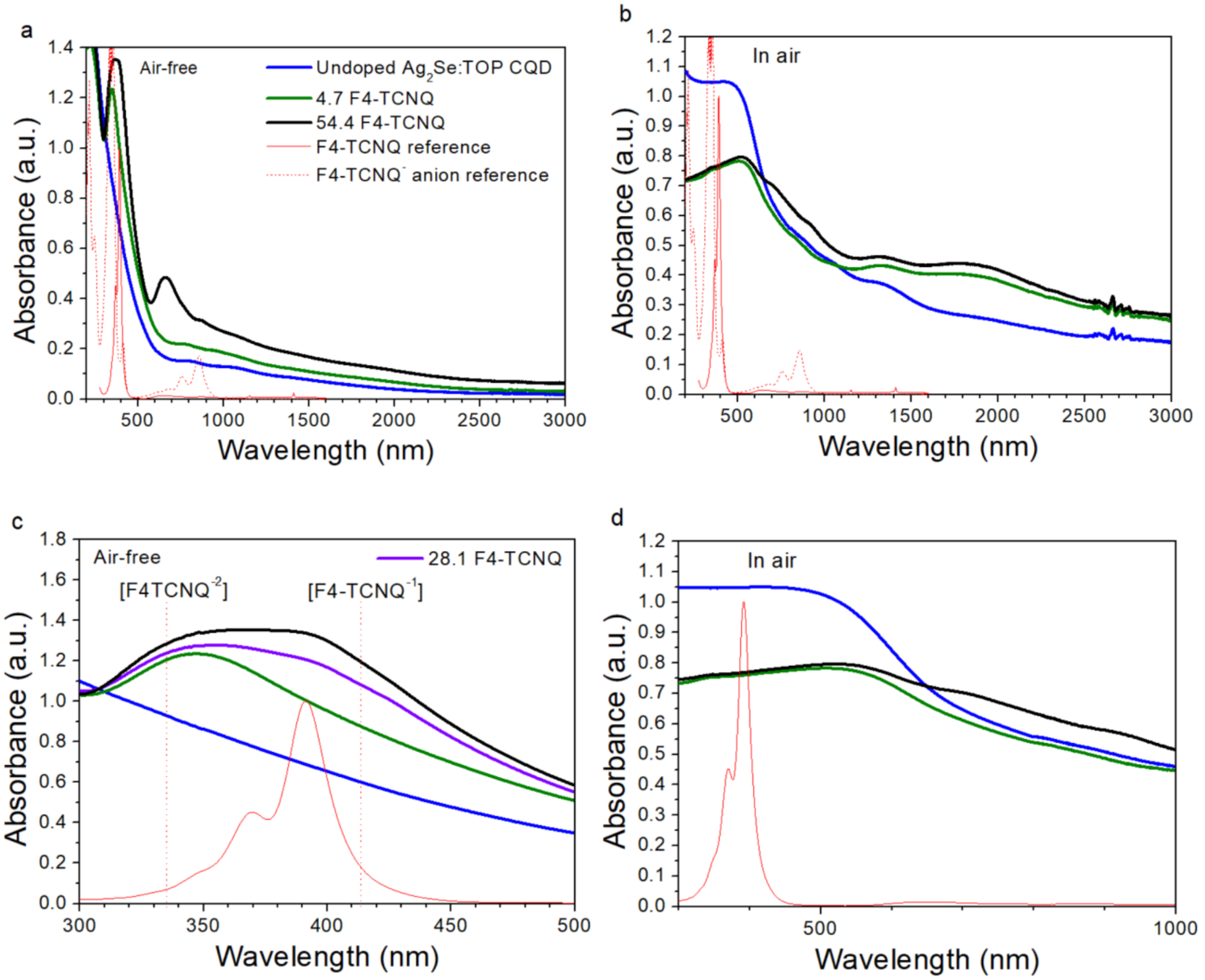

Figure S.8. Absorbance spectra in the visible/NIR/SWIR range for MWIR Ag_2Se:TOP CQDs before and after exposure to 1 mg/mL F4-TCNQ in IPA under air-free (a) and ambient (b) conditions. c) Near UV range under air-free conditions showing indication of dianion ($F4\text{-}TCNQ^{2-}$) peak at low doping concentrations. d) Near UV, visible and NIR range under ambient conditions.

Figure S.9 shows the effect of brief air-exposure for a Ag_2Se:TOP CQD thin-film doped under air-free conditions. No significant change was observed due to air-exposure, contrary to what has been observed for CQDs exposed to another molecular dopant.[12]

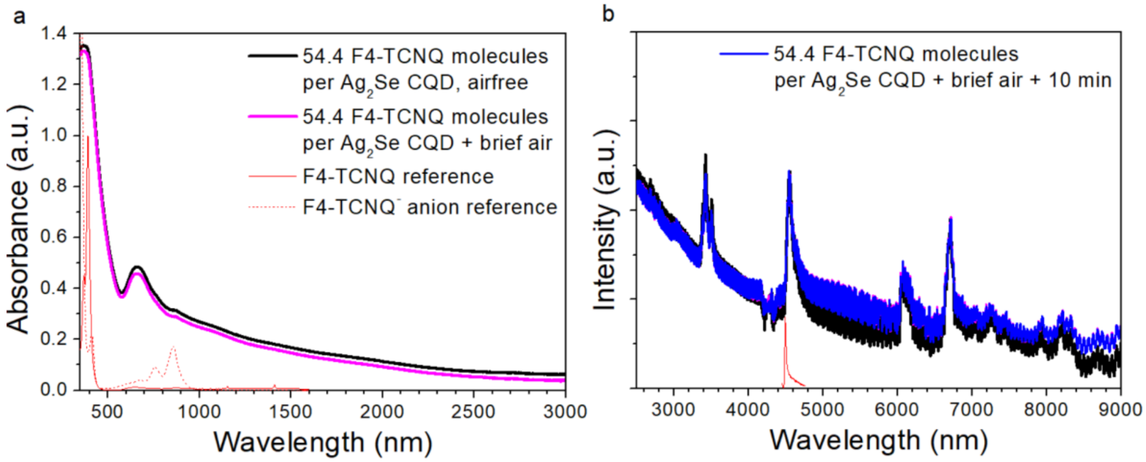

Figure S.9. Absorbance data for Ag_2Se:TOP CQDs doped with 54.4 ± 33.1 F4-TCNQ molecules per Ag_2Se CQD under air-free conditions (black solid curve) followed by brief exposure to air (Immediately after: magenta solid curve. 10 min after brief air exposure: blue solid curve). Solid and dotted red curves are reference spectra for F4-TCNQ and F4-TCNQ$^-$ monoanion, respectively. No significant change was observed due to air exposure. (a) Vis/NIR/SWIR. (b) MWIR-LWIR. The blue curve completely masks the magenta curve.

Figure S.10 shows the effect of F4-TCNQ doping on ligand exchanged MWIR-active Ag_2Se:EDT CQD films under air-free conditions. In the visible/NIR/SWIR range (Figure S.10a), a weak F4-TCNQ$^-$ monoanion peak ~876 nm, as well as evidence of charge-neutral F4-TCNQ ~400 nm/~650 nm is observed. In the MWIR range (Figure S.10b), complete quenching of the MWIR peak ~5 μm is observed at a dopant concentration of 4.7 ± 3.1 F4-TCNQ molecules per Ag_2Se CQD, with minimal shifting of the F4-TCNQ nitrile stretch, indicating a different doping mechanism than ICT.

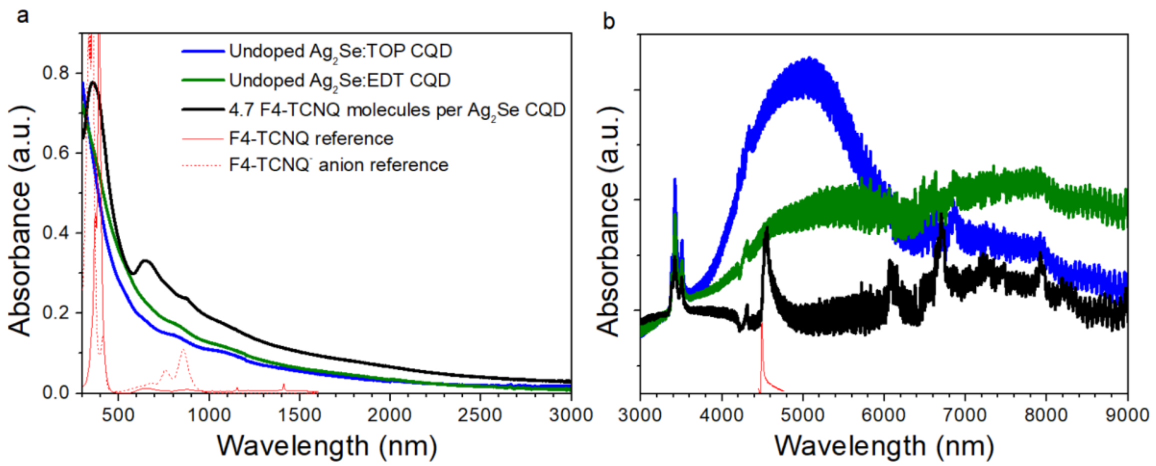

Figure S.10. Absorbance data for Ag_2Se:EDT CQDs with dropwise addition of 3.6mM F4TCNQ in IPA under air-free conditions. Blue curves: undoped Ag_2Se:TOP CQDs. Green curves: undoped, ligand exchanged, Ag_2Se:EDT CQDs. Black curves: CQDs with a doping level of 4.7 ± 3.1 F4-TCNQ molecules per Ag_2Se CQD. Solid and dotted red curves illustrate reference spectra for F4-TCNQ and F4-TCNQ$^-$ monoanion, respectively. (a) Vis/NIR/SWIR: Anion peak at 876 nm, indication of excess F4-TCNQ, Ag_2Se NIR features conserved. (b) MWIR-LWIR: Partial quenching ~5 μm after ligand exchange from TOP to EDT. Complete quenching of ~5 μm peak at ~4.7 F4-TCNQ molecules per Ag_2Se CQDs. No shifting of nitrile stretch indicates no anion formation. Peaks ~3500 nm are due to C-H stretch in ligands. Curves have been adjusted in the y-direction to better visualize quenching.

Figure S.11 shows the effect of F4-TCNQ doping on ligand exchanged MWIR-active Ag_2Se:EDT CQD films under ambient conditions. In the visible/NIR/SWIR range (Figure S.11a), no evidence of charge-neutral F4-TCNQ or F4-TCNQ$^-$ monoanion peaks are observed. A new feature at ~1100 nm appears after ligand exchange from TOP to EDT, which red-shifts along with a feature at ~500 nm with increasing F4-TCNQ doping. In the MWIR range (Figure S.11b), complete quenching of the MWIR peak ~5 μm is observed at a dopant concentration of ~128 F4-TCNQ molecules per Ag_2Se CQD, with shifting of the F4-TCNQ nitrile stretch with increasing dopant concentration, indicating partial ICT.

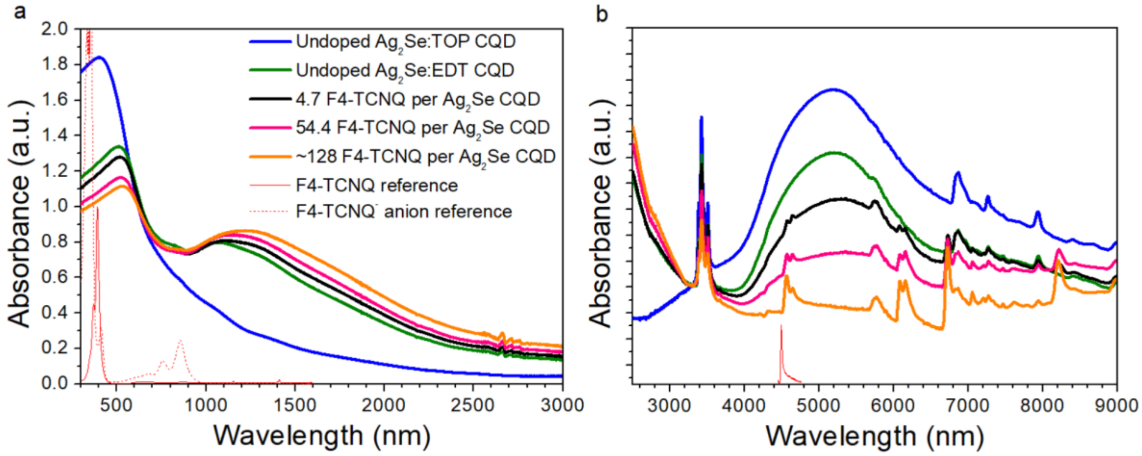

Figure S.11. Absorbance data for Ag_2Se:EDT with dropwise addition of 3.6mM F4-TCNQ in IPA under ambient conditions. Blue curves: undoped Ag_2Se:TOP CQDs. Green curves: undoped, ligand exchanged Ag_2Se:EDT CQDs. Black curves: CQDs with a doping level of 4.7 ± 3.1 F4-TCNQ molecules per Ag_2Se CQD. Magenta curves: CQDs with a doping level of 54.4 ± 33.1 F4-TCNQ molecules per Ag_2Se CQDs. Orange curves: CQDs with a doping level of ~128 ± 78 F4-TCNQ molecules per Ag_2Se CQD. The latter doping level was estimated based on the trend in doping concentration in Figure S.3. Solid and dotted red curves illustrate reference spectra for F4-TCNQ and F4-TCNQ$^-$ monoanion, respectively. (a) Vis/NIR/SWIR: No anion peak. Red-shifting of features at ~500 nm and ~1100 nm with increasing dopant concentration. (b) MWIR-LWIR: Partial quenching of ~5 μm peak after ligand exchange. Complete quenching of ~5 μm peak at ~128 F4-TCNQ molecules per Ag_2Se CQDs. Shifting of nitrile stretch indicates anion formation. Peaks ~3500 nm are due to C-H stretch in ligands. Curves have been adjusted in the y-direction to better visualize quenching.

Figure S.12 shows the effect of F4-TCNQ doping on ligand exchanged MWIR-active Ag_2Se:EDT CQD films through soaking in 0.0036M F4-TCNQ in IPA under air-free conditions. In the visible/NIR/SWIR range (Figure S.12a), evidence of excess F4-TCNQ is observed after doping, but no F4-TCNQ$^-$ monoanion peaks. In the MWIR range (Figure S.12b), complete quenching of the MWIR peak ~5 μm is observed after 20 sec soaking in F4-TCNQ solution, with minimal shifting of the F4-TCNQ nitrile stretch, indicating a different doping mechanism than ICT.

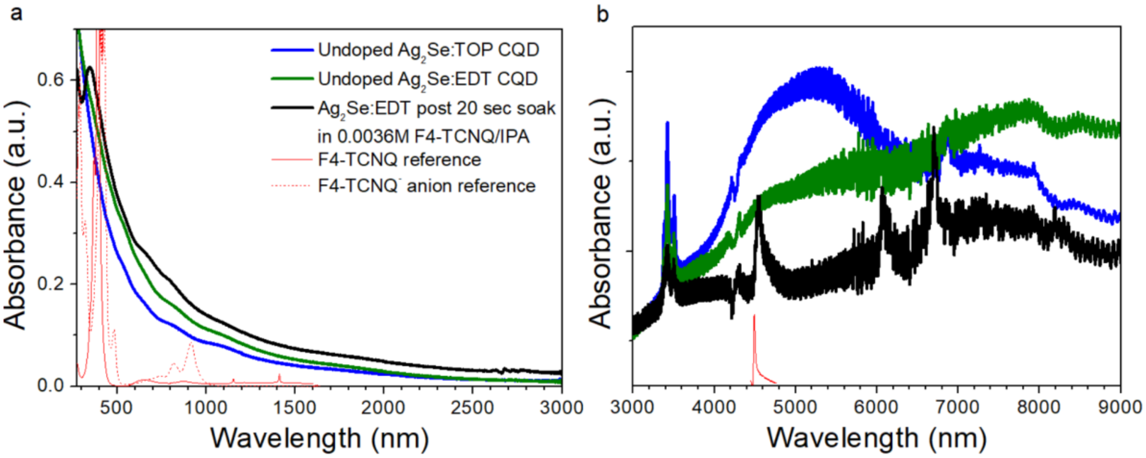

Figure S.12. Absorbance data for Ag_2Se:EDT CQDs soaked in 0.0036M F4-TCNQ in IPA under air-free conditions. (a) Vis/NIR/SWIR: No F4-TCNQ$^-$ monoanion peaks, indication of excess F4-TCNQ, Ag_2Se NIR peaks conserved. (b) MWIR-LWIR: Partial quenching ~5 μm after ligand exchange from TOP to EDT. Complete quenching of ~5 μm peak after 20 sec soaking in F4-TCNQ. No shifting of nitrile stretch indicates no anion formation. Peaks ~3500 nm are due to C-H stretch in ligands. Curves have been adjusted in the y-direction to better visualize quenching.

Figure S.13 and S.14 show the doping effect on MWIR-active Ag_2Se:TOP CQD and ligand exchanged MWIR-active Ag_2Se:EDT CQD films, respectively, through dropwise addition of 0.0036M F4-TCNQ in acetone under air-free conditions. In the visible/NIR/SWIR range (Figure S.13a and S.14a), evidence of excess F4-TCNQ as well as a slight F4-TCNQ$^-$ monoanion peak ~876 nm are observed after doping. In the MWIR range (Figure S.13b and S.14b), complete quenching of the MWIR peak ~5 μm is observed at ~5 F4-TCNQ molecules per Ag_2Se CQD, with minimal shifting of the F4-TCNQ nitrile stretch, indicating a different doping mechanism than ICT. Acetone appears to have a higher solubility towards F4-TCNQ than IPA.

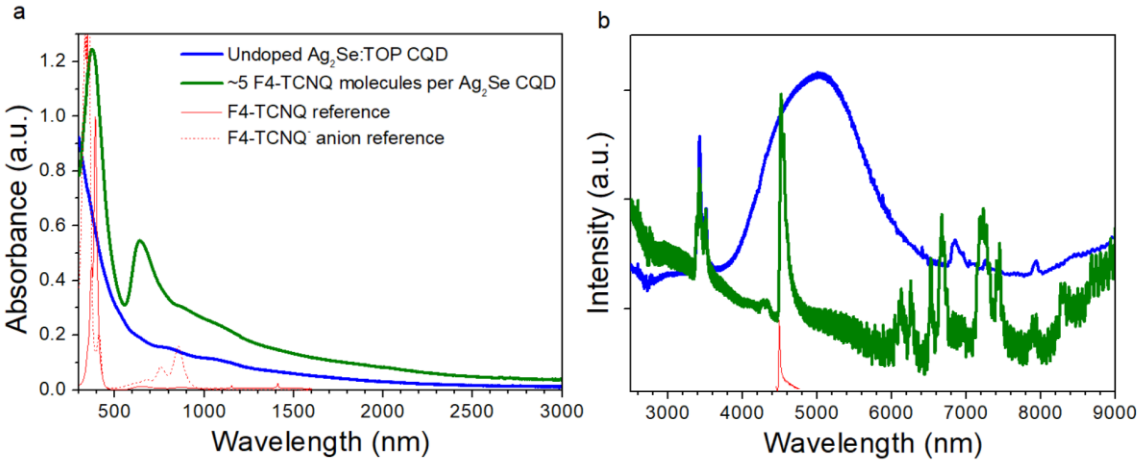

Figure S.13. Absorbance data for Ag_2Se:TOP with dropwise addition of 0.0036M F4-TCNQ in acetone under air-free conditions. Spectra for undoped Ag_2Se:TOP CQDs are shown as blue curves, and spectra for CQDs with a doping level of ~5 ± 3 F4-TCNQ molecules per Ag_2Se CQD are shown as green curves. The doping level was estimated based on the trend in doping concentration in Figure S.3. Solid and dotted red curves illustrate reference spectra for F4-TCNQ and F4-TCNQ$^-$ monoanion, respectively. (a) Vis/NIR/SWIR: Slight anion peak at ~876 nm. Excess F4-TCNQ. Slight "dent" ~2000 nm. (b) MWIR-LWIR: Complete quenching of ~5 µm peak at ~5 F4-TCNQ molecules per Ag_2Se CQDs. No shifting of nitrile stretch indicates no anion formation. Peaks ~3500 nm are due to C-H stretch in ligands. Curves have been adjusted in the y-direction to better visualize quenching.

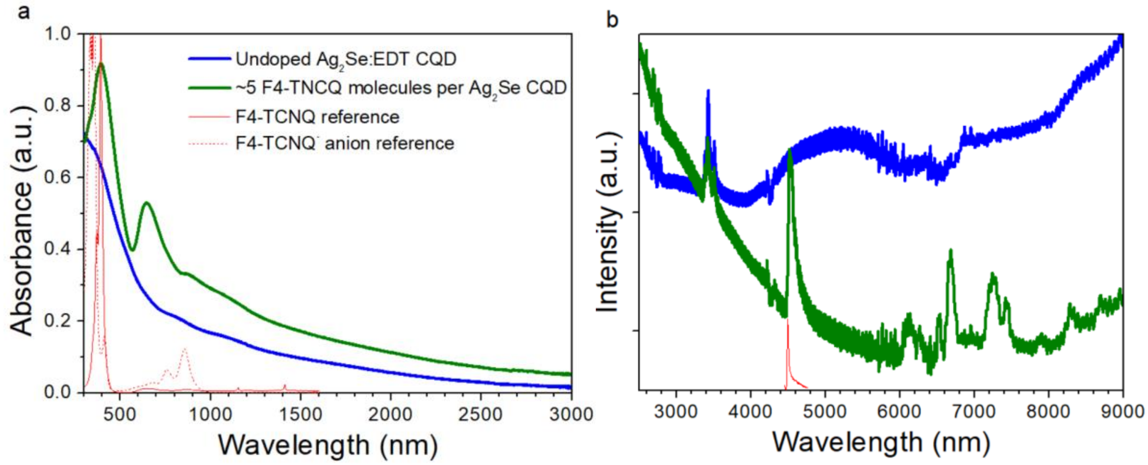

Figure S.14. Absorbance data for Ag_2Se:EDT with dropwise addition of 0.0036M F4-TCNQ in acetone under air-free conditions. Spectra for undoped Ag_2Se:EDT CQDs are shown as blue curves, and spectra for CQDs with a doping level of ~5 ± 3 F4-TCNQ molecules per Ag_2Se CQD are shown as green curves. The doping level was estimated based on the trend in doping concentration in Figure S.3. Solid and dotted red curves illustrate reference spectra for F4-TCNQ and F4-TCNQ$^-$ monoanion, respectively. (a) Vis/NIR/SWIR: Slight anion peak ~876 nm. Excess F4-TCNQ. (b) MWIR-LWIR: Complete quenching of ~5 µm peak at ~5 F4-TCNQ molecules per Ag_2Se CQD. No shifting of nitrile stretch indicates no anion formation. Peaks ~3500 nm are due to C-H stretch in ligands. Curves have been adjusted in the y-direction to better visualize quenching.

Figure S.15 shows the doping effect on ligand exchanged MWIR-active Ag_2Se:EDT CQD films through dropwise addition of 0.00058M F4-TCNQ in acetone under air-free conditions. In the visible/NIR/SWIR range (Figure S.15a), evidence of excess F4-TCNQ as well as a slight F4-TCNQ$^-$ monoanion peak ~876 nm are observed after doping. In the MWIR range (Figure S.15b), complete quenching of the MWIR peak ~5 μm is observed at ~1 F4-TCNQ molecules per Ag_2Se CQD, with minimal shifting of the F4-TCNQ nitrile stretch, indicating a different doping mechanism than ICT.

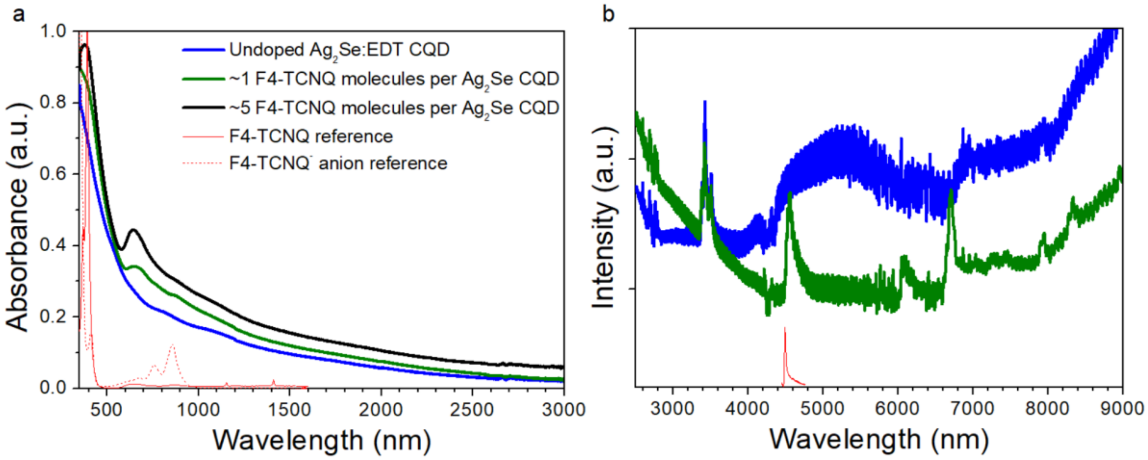

Figure S.15. Absorbance data for Ag_2Se:EDT with dropwise addition of 0.00058M F4-TCNQ in acetone under air-free conditions. Spectra for undoped Ag_2Se:EDT CQDs are shown as blue curves, spectra for CQDs with a doping level of ~1 ± 0.5 F4-TCNQ molecules per Ag_2Se CQD are shown as green curves, and spectra for doping level ~5 ± 2.5 F4-TCNQ molecules per Ag_2Se CQD is shown as black curve The doping levels were estimated based on the trend in doping concentration in Figure S.3. Solid and dotted red curves illustrate reference spectra for F4-TCNQ and F4-TCNQ$^-$ monoanion, respectively. (a) Vis/NIR/SWIR: Slight anion peak ~876 nm at lowest doping concentration. Excess F4-TCNQ. (b) MWIR-LWIR: Complete quenching of ~5 μm peak at ~1 F4-TCNQ molecule per Ag_2Se CQD. No shifting of nitrile stretch indicates no anion formation. Peaks ~3500 nm are due to C-H stretch in ligands. Curves have been adjusted in the y-direction to better visualize quenching.

Section 5: Doping of bulk Ag_2Se

Doping of bulk Ag_2Se was investigated in order to decouple any nanoscale effects affecting the molecular doping of this semiconductor system by F4-TCNQ. Bulk Ag_2Se thin-films were fabricated based on a method inspired by Webber and Brutchey,[13,14] through a cation exchange process from copper (I) selenide (Cu_2Se) direct thin films. Briefly, 100 mg Cu_2Se was dissolved in 2 mL ethylenediamine and 0.2 mL 1,2-ethanedithiol, spin coated (1800 rpm, 60 sec) onto 1x1 cm clean glass substrates and annealed at 350 °C for 1 hour. The cation exchange process consisted of soaking the $Cu_{2-x}Se$ films in 0.01M silver nitrate ($AgNO_3$) in methanol for 10 min, followed by 45 sec rinse in neat methanol and drying for 2 min on a hot plate at 50 °C. The cation exchanged films were annealed at 350 °C for 30 min. Finally, 60 nm gold was thermally evaporated through a shadow mask onto all four corners of the substrates as electrical contacts. All fabrication steps took place in a N_2-filled glovebox.

The bulk film samples were exposed to 1 mg/mL F4-TCNQ in IPA or chloroform dropwise, and room temperature sheet resistance, R_{sheet}, and Seebeck coefficient, α, were measured between each new exposure. R_{sheet} was measured in a standard 4-probe van-der-Pauw setup, while the α was determined in a homemade setup using small Peltier units (CUI Inc.) to provide temperature gradients and Keithley 2400 source meters and Keithley 2000 multimeters to provide currents sources and measure voltages. Custom Labview programs were used to control the instruments and to process and save the data.

The Seebeck coefficient for the as-fabricated Ag_2Se samples was on the order of -40 μV/K and the R_{sheet} was on the order of 76 Ohm/sq. This is as expected for an unoptimized sample fabricated through this method,[14] the negative α coefficient confirming the n-type carrier transport in this semiconductor. After exposure to a single drop of 1 mg/mL F4-TCNQ in IPA, the R_{sheet} increased by 19% and the absolute value of α increased by 111%. This can be explained through the decrease in available charge carriers, n, and demonstrates the doping impact of F4-TCNQ for bulk Ag_2Se. Upon exposure to another drop of 1 mg/mL F4-TCNQ in IPA, the R_{sheet} decreased slightly, indicating that the limited surface area of bulk Ag_2Se had already been saturated with F4-

TCNQ, and that the additional IPA only served to redissolve some of the F4-TCNQ$^-$ monoanions. The R_{sheet} stayed fairly constant during storage for two days in a N_2-filled glovebox, and the R_{sheet} did not change much upon 10 sec soaking in neat IPA. Annealing at 300 °C for 30 min reversed the initial change in R_{sheet}. The relative change in R_{sheet} is shown in **Figure S.16**. F4-TCNQ has a melting point of 291 °C,[15] but has been reported to sublimate even below 200 °C,[16,17] thus annealing at 300 °C was expected to vaporize the F4-TCNQ. The bulk Ag_2Se thin-films had already been exposed to a temperature 350 °C prior to doping.

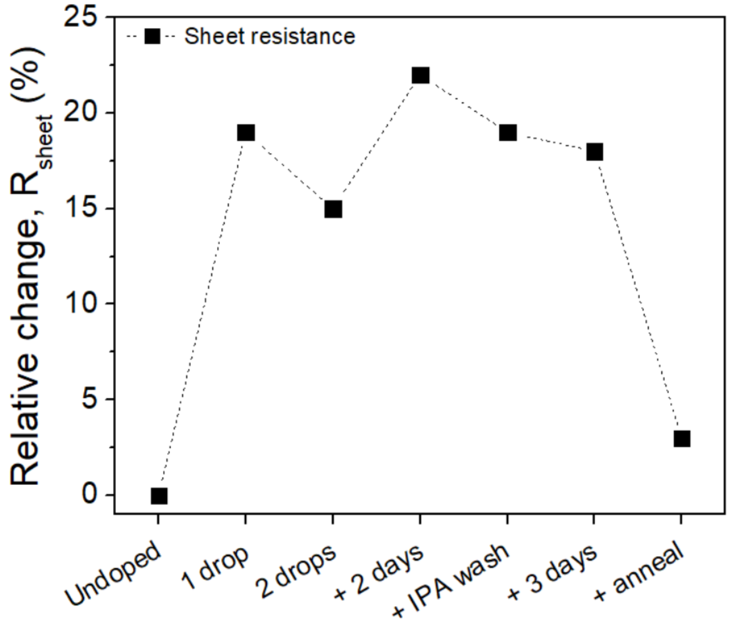

Figure S.16. Relative change in R_{sheet} for a bulk Ag_2Se thin-film on glass substrate exposed to 1 mg/mL F4-TCNQ in IPA.

The polarity of the solvent has been suggested to play an important role in the interaction between F4-TCNQ and the host material.[11] Therefore, chloroform, which has a slightly lower polarity than IPA, was utilized as a solvent in a 1 mg/mL F4-TCNQ solution. Similarly to for IPA, exposure of the Ag_2Se film to a single drop of F4-TCNQ in chloroform yields an increase in both the R_{sheet} and the Seebeck coefficient, however slightly lower than with IPA (10% and 20% for R_{sheet} and Seebeck, respectively, compared to 19% and 111% using IPA) The R_{sheet} stayed fairly stable during storage for one day in a N_2 filled glovebox, and increased further with exposure to another drop of F4-TCNQ in

chloroform the following day. Exposure to a 3rd drop of F4-TCNQ in chloroform resulted in a decrease in R_{sheet}, again demonstrating the limited surface area of bulk Ag_2Se available for surface interactions with F4-TCNQ. Relative change in R_{sheet} for bulk Ag_2Se with exposure to F4-TCNQ in chloroform is summarized in **Figure S.17**:

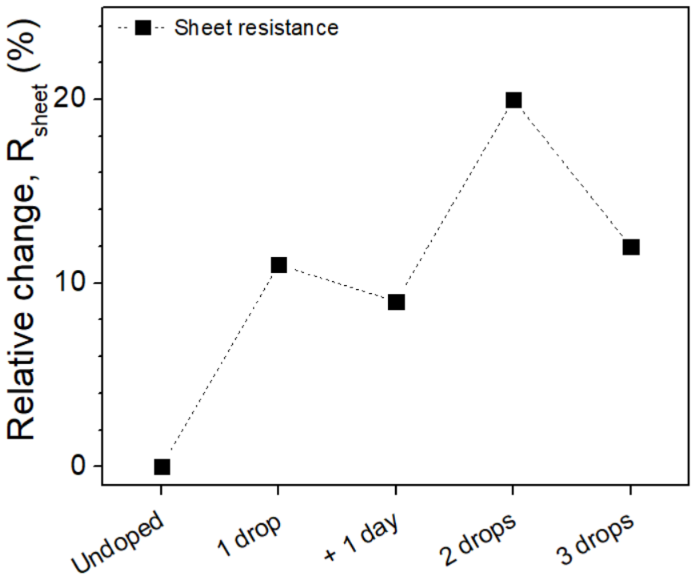

Figure S.17. Relative change in R_{sheet} for a bulk Ag_2Se thin-film on glass substrate exposed to 1 mg/mL F4-TCNQ in chloroform.

Overall, these results demonstrate that doping of Ag_2Se by F4-TCNQ is possible, not only for quantum confined nanoparticles, but also for bulk thin-films, although the doping effect might be limited by available surface area in bulk films.

Section 6: Other doping strategies

HgS CQDs are known for their stable n-type doping in the ambient,[18] with strong absorbance features in the MWIR and NIR. Jeong et al. demonstrated that the spectral response of these CQDs was sensitive to surface treatment. Exposing the CQD film to S^{2-} ions, the MWIR absorption was removed and the NIR edge red shifted, while with subsequent treatment with Hg^{2+}, the MWIR absorption was reintroduced and the NIR

edge blue shifted. The authors claim that this was the first demonstration of stabile surface doping controlled in ambient, and suggest that the doping can be attributed to rigid shifts of the energy bands with respect to the environment. Dopant concentration during the solution phase doping step was ~7 mM for both S^{2-} and Hg^{2+}.

In the report by Yarema et al.[19] the authors claim to utilize a donor-acceptor heterojunction to fabricate a lateral (near) IR photodetector. Mixing Ag_2Se CQD with [6,6]-phenyl-C_{61}-butyric acid methyl ester (PCBM) in a 1:4 weight ratio and depositing a blended film on a glass substrate with interdigitated ITO electrodes, the authors measured a responsivity of 200 mA/W at 900 nm, with photoresponse up to 1300 nm for a device with 13.5 mm^2 active area. The authors insinuate that an electron transfer is taking place between the CQDs and PCBM, but do not report any change in the spectral response of the CQDs as a result of the blending. With a reported work function of -4.2-4.3 eV for PCBM,[20] the reported valence band edge of NIR Ag_2Se of -4.9 eV by Graddage et al.,[7] and our measured E_F of Ag_2Se of -4.3 eV, the driving force for electron transfer from the CQD to PCBM seems low. Rather, it could be the improved mobility due to excess PCBM in the blend, as well as the high applied bias (100 V) that yields a working Ag_2Se sensitized photodetector with such high responsivity.

References

(1) Lhuillier, E.; Keuleyan, S.; Guyot-Sionnest, P. Optical properties of HgTe colloidal quantum dots. *Nanotechnology* **2012**, *23* (17), 175705. DOI: 10.1088/0957-4484/23/17/175705.

(2) Scimeca, M. R.; Mattu, N.; Paredes, I. J.; Tran, M. N.; Paul, S. J.; Aydil, E. S.; Sahu, A. Origin of Intraband Optical Transitions in Ag2Se Colloidal Quantum Dots. *J. Phys. Chem. C* **2021**, *125* (31), 17556-17564. DOI: 10.1021/acs.jpcc.1c05371.

(3) Sahu, A.; Khare, A.; Deng, D. D.; Norris, D. J. Quantum confinement in silver selenide semiconductor nanocrystals. *Chem Commun (Camb)* **2012**, *48* (44), 5458-5460. DOI: 10.1039/c2cc30539a.

(4) Sahu, A.; Braga, D.; Waser, O.; Kang, M. S.; Deng, D.; Norris, D. J. Solid-Phase Flexibility in Ag2Se Semiconductor Nanocrystals. *Nano Lett.* **2014**, *14* (1), 115-121. DOI: 10.1021/nl4041498.

(5) Méndez, H.; Heimel, G.; Winkler, S.; Frisch, J.; Opitz, A.; Sauer, K.; Wegner, B.; Oehzelt, M.; Röthel, C.; Duhm, S.; et al. Charge-transfer crystallites as molecular electrical dopants. *Nat. Commun.* **2015**, *6* (1), 8560. DOI: 10.1038/ncomms9560.

(6) Qu, J.; Goubet, N.; Livache, C.; Martinez, B.; Amelot, D.; Gréboval, C.; Chu, A.; Ramade, J.; Cruguel, H.; Ithurria, S.; et al. Intraband Mid-Infrared Transitions in Ag2Se Nanocrystals: Potential and Limitations for Hg-Free Low-Cost Photodetection. *J. Phys. Chem. C* **2018**, *122* (31), 18161-18167. DOI: 10.1021/acs.jpcc.8b05699.

[7] Graddage, N., Ouyang, J., Lu, J.,Chu, T.-Y., Zhang, Y., Li, Z., Wu, X., Malenfant, P. R. L., Tao, Y., Near-Infrared-II Photodetectors Based on Silver Selenide Quantum Dots on Mesoporous TiO2 Scaffolds, *ACS Appl. Nano Mater.* **2020**, 3, 12209-12217. DOI: 10.1021/acsanm.0c02686

[8] Cochran, J. E.; Junk, M. J. N.; Glaudell, A. M.; Miller, P. L.; Cowart, J. S.; Toney, M. F.; Hawker, C. J.; Chmelka, B. F.; Chabinyc, M. L. Molecular Interactions and Ordering in Electrically Doped Polymers: Blends of PBTTT and F4TCNQ. *Macromolecules* **2014**, *47* (19), 6836-6846. DOI: 10.1021/ma501547h.

(9) Li, J.; Zhang, G.; Holm, D. M.; Jacobs, I. E.; Yin, B.; Stroeve, P.; Mascal, M.; Moulé, A. J. Introducing Solubility Control for Improved Organic P-Type Dopants. *Chem. Mater.* **2015**, *27* (16), 5765-5774. DOI: 10.1021/acs.chemmater.5b02340.

(10) Misseeuw, L.; Krajewska, A.; Pasternak, I.; Ciuk, T.; Strupinski, W.; Reekmans, G.; Adriaensens, P.; Geldof, D.; Blockhuys, F.; Van Vlierberghe, S.; et al. Optical-quality controllable wet-chemical doping of graphene through a uniform, transparent and low-roughness F4-TCNQ/MEK layer. *RSC Adv.* **2016**, *6* (106), 104491-104501, 10.1039/C6RA24057G. DOI: 10.1039/C6RA24057G.

(11) Stanfield, D. A.; Wu, Y.; Tolbert, S. H.; Schwartz, B. J. Controlling the Formation of Charge Transfer Complexes in Chemically Doped Semiconducting Polymers. *Chem. Mater.* **2021**, *33* (7), 2343-2356. DOI: 10.1021/acs.chemmater.0c04471.

(12) Koh, W.-k.; Koposov, A. Y.; Stewart, J. T.; Pal, B. N.; Robel, I.; Pietryga, J. M.; Klimov, V. I. Heavily doped n-type PbSe and PbS nanocrystals using ground-state charge transfer from cobaltocene. *Sci. Rep.* **2013**, *3* (1), 2004. DOI: 10.1038/srep02004.

(13) Webber, D. H.; Brutchey, R. L. Alkahest for V2VI3 Chalcogenides: Dissolution of Nine Bulk Semiconductors in a Diamine-Dithiol Solvent Mixture. *J. Am. Chem. Soc.* **2013**, *135* (42), 15722-15725. DOI: 10.1021/ja4084336.

(14) Chen, N.; Scimeca, M. R.; Paul, S. J.; Hafiz, S. B.; Yang, Z.; Liu, X.; Yang, F.; Ko, D.-K.; Sahu, A. High-performance thermoelectric silver selenide thin films cation exchanged from a copper selenide template. *Nanoscale Adv.* **2020**, *2* (1), 368-376. DOI: 10.1039/C9NA00605B.

(15) Ossila. *F4TCNQ*. Ossila Ltd, **2023.** https://www.ossila.com/en-us/products/f4TCNq (accessed 2023 November)

(16) Jacobs, I. E.; Moulé, A. J. Controlling Molecular Doping in Organic Semiconductors. *Adv. Mater.* **2017**, *29* (42), 1703063. DOI: 10.1002/adma.201703063.

(17) DiTusa, M. F.; Grocke, G. L.; Ma, T.; Patel, S. N. Probing the evolution of conductivity and structural changes in vapor-F4TCNQ doped P3HT. *Mol. Syst. Des. Eng.* **2022**, *7* (7), 788-797. DOI: 10.1039/D1ME00192B.

(18) Jeong, K. S., Deng, Z., Keuleyan, S., Liu, H., Guyot-Sionest, P., Air-stable n-Doped Colloidal HgS Quantum Dots, *J. Phys. Chem. Lett.* **2014**, 5, 1139-1143. DOI 10.1021/jz500436x

(19) Yarema, M., Pichler, S., Sytnyk. M., Seyrkammer, R., Lechner, R. T., Fritz-Popovski, G., Jarzab, D., Szendrei, K., Resel, R., Korovyanko, O., Loi, M. A., Paris, O., Hesser, G., Heiss, W., *ACS Nano* **2011**, 5 (5), 3758-3765. DOI 10.1021/nn2001118

(20) Soci, C., Hwang, I.-W., Moses, D., Zhu, Z., Waller, D., Gaudiana, R., Brabec, C. J., Heeger, A. J., *Adv. Funct. Mater.* **2007**, 17, 632-636. DOI 10.1002/adfm.200600199
